# Circulating Neurotransmitters, Neuropeptides, and Neuroactive Mediators in Gastric Cancer: A Systematic Review of Diagnostic, Prognostic, and Clinicopathological Evidence

**DOI:** 10.3390/cancers18213562

**Published:** 2026-11-06

**Authors:** Bogdan Cristian Albu, Ancuța-Ramona Camen, Mihail Virgil Boldeanu, Mohamed-Zakaria Assani, Carmen Valeria Albu, Lidia Boldeanu, Elena Albu, Dragos Ene, Mihai Călin Ciorbagiu, Emil Moraru, Cecil Sorin Mirea

**Affiliations:** 1 Doctoral School, University of Medicine and Pharmacy of Craiova, 200349 Craiova, Romania; cristian.albu@umfcv.ro (B.C.A.); mohamed.assani@umfcv.ro (M.-Z.A.); 2 Department of Occupational Medicine, University of Medicine and Pharmacy of Craiova, 200349 Craiova, Romania; ancuta.boicea@umfcv.ro; 3 Department of Immunology, Faculty of Medicine, University of Medicine and Pharmacy of Craiova, 200349 Craiova, Romania; mihail.boldeanu@umfcv.ro; 4 Research Center of Periodontal-Systemic Interactions, University of Medicine and Pharmacy of Craiova, 200349 Craiova, Romania; 5 Department of Neurology, Faculty of Medicine, University of Medicine and Pharmacy of Craiova, 200349 Craiova, Romania; carmen.albu@umfcv.ro; 6 Department of Microbiology, Faculty of Medicine, University of Medicine and Pharmacy of Craiova, 200349 Craiova, Romania; 7 Department 10—General Surgery, “Carol Davila” University of Medicine and Pharmacy, 050474 Bucharest, Romania; dragos.ene@umfcd.ro; 8 Department of Surgical Semiology, Faculty of Medicine, University of Medicine and Pharmacy of Craiova, 200349 Craiova, Romania; mihai.ciorbagiu@umfcv.ro (M.C.C.); emil.moraru@umfcv.ro (E.M.); cecil.mirea@umfcv.ro (C.S.M.)

**Keywords:** gastric cancer, circulating biomarkers, neurotransmitters, neuropeptides, neuroactive mediators, galanin, ProGRP, serotonin, neurotensin, dopamine, neural–tumor interactions

## Abstract

Background/Objectives: Neural–tumor interactions are increasingly recognized in gastric cancer (GC), yet their relationship with circulating neuroactive mediators remains incompletely understood. This systematic review aimed to synthesize human evidence on circulating neurotransmitters, neuropeptides, and related mediators, assess their diagnostic, clinicopathological, treatment-associated, and prognostic relevance, and integrate clinical findings within a translational framework of neuro–tumor signaling. Methods: PubMed/MEDLINE, Scopus, and Web of Science Core Collection were systematically searched following PRISMA 2020 guidelines. Eligible studies assessed circulating neuroactive mediators in patients with GC. Clinical findings were synthesized by evidence domain and contextualized using complementary tumor-level receptor and signaling evidence. Results: Twenty-four publications representing 23 independent cohorts were included. Replicated circulating alterations were identified for galanin, motilin, and cholecystokinin, although gastrointestinal physiology and treatment-related changes complicated their interpretation. Progastrin-releasing peptide demonstrated diagnostic discrimination in a single study (AUC 0.713; sensitivity 85.9%; specificity 81.2%) and associations with clinicopathological characteristics. Dopaminergic and catecholamine-related alterations suggested systemic neuroendocrine involvement, while higher normetanephrine was associated with poorer survival in univariable analysis. Complementary mechanistic evidence implicated serotonergic, substance P/NK-1R, and neurotensin/NTSR1 pathways in tumor-associated signaling. The translational evidence map highlighted three overlapping biological dimensions: tumor-associated receptor-mediated signaling, systemic neuroendocrine and stress adaptations, and gastrointestinal physiological responses. However, small cohorts, heterogeneous biological matrices, historical assay variability, and limited independent validation restricted clinical interpretation. Conclusions: Circulating neuroactive mediators represent a biologically plausible but insufficiently validated biomarker domain in GC. Their clinical translation requires standardized prospective studies integrating circulating measurements with matched tumor biology, clinically relevant comparator populations, longitudinal outcomes, and independent validation. Pathway-oriented multimarker approaches may ultimately provide more informative neuroactive signatures than individual mediators.

## Simple Summary

Gastric cancer remains a major health challenge, highlighting the need for better blood-based markers that could support diagnosis, disease monitoring, and prognosis. Chemicals involved in communication between nerve cells, including neurotransmitters and neuropeptides, may also influence cancer development and progression. This systematic review examined 24 published studies investigating these substances in the blood of patients with gastric cancer. Several compounds, particularly galanin, a precursor of gastrin-releasing peptide, and motilin, showed potentially relevant changes associated with cancer presence, disease characteristics, or treatment. However, findings varied considerably between studies, partly because of differences in patient populations and laboratory methods. Although these substances may provide new insights into the relationship between the nervous system and gastric cancer, none are currently sufficiently validated for routine clinical use. Larger, carefully designed studies are needed to establish their potential value as complementary blood-based biomarkers.

## 1. Introduction

Gastric cancer (GC) remains a major global health burden despite advances in prevention, endoscopic detection, molecular classification, surgery, and systemic therapy. According to the most recent GLOBOCAN estimates, nearly one million new cases of stomach cancer and more than 640,000 related deaths occurred worldwide in 2024, making it the sixth most commonly diagnosed cancer and the fifth leading cause of cancer-related mortality [1]. Its clinical course is highly heterogeneous, reflecting differences in tumor location, histological phenotype, molecular background, stage at diagnosis, host characteristics, and treatment response. Although established circulating markers such as carcinoembryonic antigen (CEA), carbohydrate antigen 19-9 (CA19-9), and CA72-4 may provide complementary information in selected patients, their limited sensitivity and specificity preclude their use as stand-alone tools for early diagnosis or comprehensive disease stratification. Consequently, substantial interest remains in identifying circulating biomarkers that capture additional dimensions of GC biology and may complement conventional tumor markers and tissue-based molecular profiling.

An increasingly recognized dimension of cancer biology is the bidirectional interaction between malignant cells and the nervous system. The stomach is densely innervated by intrinsic enteric neurons and extrinsic autonomic and sensory pathways, creating a particularly complex environment in which neural, endocrine, immune, stromal, and epithelial signals converge. During gastric carcinogenesis, this physiological neuroepithelial network may undergo substantial remodeling through altered innervation, perineural interactions, neurogenesis, and axonogenesis [2,3]. Conversely, tumor cells and components of the tumor microenvironment may produce, respond to, or metabolically modify neuroactive mediators. These interactions can influence proliferation, survival, migration, invasion, angiogenesis, immune regulation, cellular metabolism, and therapeutic resistance [2,3]. Thus, neurotransmitters and neuropeptides should no longer be considered exclusively as neuronal messengers, but also as components of intercellular signaling networks operating within and beyond the tumor microenvironment.

Several neuroactive pathways have been implicated in GC. Researchers have investigated monoaminergic signaling involving serotonin, dopamine (DA), and catecholamines; peptidergic pathways involving galanin (GAL), substance P (SP), neuropeptide Y (NPY), neurotensin (NT), vasoactive intestinal peptide (VIP), and gastrin-releasing peptide (GRP); and additional gut–brain mediators at different experimental and clinical levels [2,3,4]. Their biological effects may involve classical receptor-dependent pathways, including cAMP, Ca^2+^-dependent signaling, PKC, MAPK/ERK, PI3K/AKT, and STAT3, as well as more recently recognized receptor-independent mechanisms. Particularly notable is recent evidence linking serotonin to protein serotonylation in GC. Bai et al. reported increased plasma 5-hydroxytryptamine (5-HT) in patients with GC and later showed that transglutaminase 2 (TGM2)-mediated serotonylation stabilizes glutathione peroxidase 4 (GPX4), promoting ferroptosis resistance and gastric tumor growth [5]. Such findings reinforce the concept that neuroactive mediators may simultaneously represent measurable systemic alterations and functionally relevant components of tumor biology.

However, mechanistic involvement in GC does not necessarily translate into utility as a circulating biomarker. Existing research spans markedly different biological compartments, including tumor tissue, adjacent gastric mucosa, peripheral blood mononuclear cells, serum, plasma, whole blood, gastric juice, experimental models, and cancer cell lines. This heterogeneity matters for neuroactive mediators because circulating concentrations may be influenced by neuronal release, platelet storage, endocrine secretion, gastrointestinal physiology, immune-cell activity, systemic stress responses, nutritional status, medications, and pre-analytical handling. Moreover, serum, plasma, and whole-blood measurements are not necessarily interchangeable. Consequently, tissue expression or receptor activation cannot be assumed to predict the direction or magnitude of a corresponding circulating alteration. The clinical biomarker question therefore requires evaluation independently from the broader mechanistic literature.

Clinical interest in circulating neuroactive mediators in gastric cancer has emerged from the recognition that neural signaling is not confined to the tumor microenvironment but may also be reflected in systemic biochemical alterations. Neurotransmitters, neuropeptides, and related metabolites support physiological processes such as gastrointestinal motility, secretion, vascular regulation, immune responses, and neuroendocrine homeostasis. Their circulating concentrations may therefore reflect complex interactions among tumor-associated signaling, host responses, and gastrointestinal physiology. This biological complexity supports investigating these compounds as potential biomarkers while also highlighting key challenges in interpreting their clinical significance.

A key consideration is distinguishing the biological activity of a neuroactive pathway from the measurable concentration of its components in peripheral blood. Local production, receptor expression, enzymatic degradation, tissue uptake, and systemic clearance may influence circulating concentrations independently of tumor-specific signaling. Moreover, the clinical interpretation of these mediators depends on the biochemical identity of the measured analyte. Classical neurotransmitters, neuropeptides, peptide precursors, and catecholamine metabolites represent related but biologically distinct molecular categories, with different physiological sources, metabolic characteristics, and analytical requirements. Consequently, alterations in circulating concentrations cannot automatically be interpreted as direct indicators of pathway activation within gastric tumors.

The potential clinical applications of circulating neuroactive mediators extend beyond distinguishing patients with GC from individuals without malignancy. As blood-based molecular indicators, these mediators could complement established endoscopic, histopathological, and imaging approaches by providing information on systemic neuroendocrine and neuroimmune alterations that may not be directly captured by morphological assessment. Their investigation may contribute to identifying biologically distinct patient subgroups, characterizing associations with tumor aggressiveness and disease progression, and evaluating longitudinal changes following surgical or systemic treatment. Furthermore, the involvement of selected neuroactive signaling pathways in tumor–microenvironment interactions may provide a biological rationale for investigating novel therapeutic targets, although circulating concentrations do not necessarily reflect intratumoral pathway activity or establish therapeutic targetability. Importantly, the potential use of these mediators for risk stratification, treatment selection, or therapeutic monitoring remains exploratory and requires standardized analytical procedures, assessment of relevant confounders, and prospective validation demonstrating incremental clinical value beyond conventional tumor markers and established clinicopathological parameters.

Despite these observations, clinical evidence remains fragmented across individual mediators, blood matrices, analytical platforms, and clinical endpoints. Most candidate markers have been evaluated in only one or a few relatively small cohorts, and independent external validation is uncommon. Furthermore, the direction of circulating alterations may not always be consistent across studies. This issue is particularly relevant for serotonin, for which historical whole-blood measurements and contemporary plasma metabolomic investigations have yielded apparently divergent patterns. Such discrepancies may reflect differences in biological matrix, platelet contribution, sampling and processing conditions, assay methodology, disease stage, or other clinical and pre-analytical factors rather than true biological contradiction. Therefore, systematic interpretation requires attention not only to the mediator’s identity but also to how and where it was measured.

Recent reviews have substantially advanced understanding of neural–cancer crosstalk and neurotransmitter-mediated regulation in GC [2,3]. In addition, a systematic review has specifically examined the role of DA in gastric carcinogenesis, integrating experimental and mechanistic evidence concerning dopaminergic signaling [4]. However, these approaches primarily address the biological involvement of neural signaling in tumor initiation and progression rather than systematically evaluating neuroactive compounds measured in the circulation of patients with GC. To our knowledge, the dispersed human evidence concerning circulating neurotransmitters, neuropeptides, their precursors, and closely related neuroactive metabolites has not been comprehensively synthesized across diagnostic, clinicopathological, treatment-monitoring, and prognostic domains.

Therefore, the present systematic review aimed to identify and critically synthesize human studies evaluating circulating neurotransmitters, neuropeptides, and related neuroactive mediators in GC. Specifically, we sought to (i) characterize differences in circulating concentrations between patients with GC and relevant comparator groups; (ii) examine associations with tumor size, invasion, nodal and distant metastasis, TNM stage, histological characteristics, and other clinicopathological variables; (iii) evaluate changes following surgical or systemic treatment and evidence concerning recurrence or survival; and (iv) integrate circulating findings with selected tissue-level and mechanistic evidence to identify biologically supported signals and major translational gaps. By distinguishing circulating biomarker evidence from tissue and experimental observations, this review seeks to define the current clinical evidence base and provide a framework for the prospective validation of neuroactive mediators as complementary biomarkers in GC.

## 2. Materials and Methods

### 2.1. Review Design and Reporting Framework

This systematic review aimed to identify and synthesize clinical evidence on circulating neurotransmitters, neuropeptides, and related neuroactive mediators in patients with GC. The systematic review was reported in accordance with the Preferred Reporting Items for Systematic Reviews and Meta-Analyses (PRISMA) 2020 guidelines, and the completed PRISMA 2020 checklist is provided in Supplementary Table S1 [6].

The review question was formulated as follows: In patients with GC, which circulating neurotransmitters, neuropeptides, and related neuroactive mediators have been investigated, and what evidence supports their diagnostic, clinicopathological, treatment-monitoring, or prognostic significance?

Following completion of the review, its methodology was retrospectively registered in the Open Science Framework (OSF Registries; https://osf.io/2ym5n (accessed on 8 October 2026)). Registration occurred after completion of the literature search, study selection, data extraction, and evidence synthesis and therefore does not constitute prospective protocol registration. Consequently, the registration record cannot independently establish whether the eligibility criteria, search strategy, outcome domains, or synthesis methods remained unchanged throughout the review process. In the absence of a prospectively registered protocol, potential deviations from the initial methodological plan cannot be formally assessed against a time-stamped prespecified protocol. To mitigate this limitation, the present manuscript provides explicit descriptions of the eligibility criteria, information sources, search procedures, study-selection framework, data extraction, and synthesis approach, together with the corresponding supplementary documentation. Nevertheless, retrospective registration does not eliminate the possibility of post hoc methodological decisions and should be considered a limitation when evaluating the transparency and reproducibility of the review.

### 2.2. Information Sources and Search Strategy

We systematically searched PubMed/MEDLINE, Scopus, and Web of Science Core Collection from database inception to 27 September 2026, without publication-date or language restrictions. The search strategies combined controlled vocabulary, where available, with free-text terms related to gastric malignancy, neurotransmitters, neuropeptides, and related neuroactive mediators, including both broad terminology and individual compound names.

The database searches identified 5202 records before deduplication: 1676 from PubMed/MEDLINE, 2455 from Scopus, and 1071 from Web of Science Core Collection. Complete database-specific search syntaxes, search dates, restrictions, and retrieval counts are provided in Supplementary Table S2.

We additionally screened the reference lists of eligible studies and relevant reviews to identify potentially relevant publications not retrieved through the electronic searches. Targeted supplementary searches using individual mediator names were performed when necessary to identify historical studies or clarify ambiguous indexing. These supplementary searches did not modify the predefined eligibility criteria.

### 2.3. Eligibility Criteria

Studies were eligible when they met all of the following criteria: (1) original human research; (2) inclusion of adult patients with histologically or clinically diagnosed gastric cancer, gastric carcinoma, or gastric adenocarcinoma; (3) measurement of at least one neurotransmitter, neuropeptide, neuropeptide precursor, catecholamine metabolite, or closely related neuroactive mediator in serum, plasma, or whole blood; and (4) reporting of extractable information relevant to at least one prespecified clinical domain: comparison with healthy or non-cancer controls, diagnostic performance, clinicopathological characteristics, treatment-associated changes, recurrence, progression, or survival.

Eligible neuroactive compounds included classical or monoaminergic neurotransmitters; catecholamine metabolites; neuropeptides; endogenous opioid peptides; the GRP pathway; and selected gut–brain peptides with recognized neuroactive signaling functions. Studies measuring multiple analytes were eligible when at least one prespecified neuroactive mediator could be individually identified and its GC-specific results extracted.

We imposed no minimum sample size because the literature includes historical and exploratory studies in rare or poorly investigated neuroactive pathways. We retained small studies to characterize the full evidence base but interpreted them in light of their methodological limitations and risk of bias.

Studies were excluded if they: (1) were animal, cell-line, or other exclusively experimental studies; (2) assessed the relevant mediator only in tumor tissue, adjacent mucosa, peripheral blood mononuclear cells, gastric juice, or another non-circulating compartment without extractable serum, plasma, or whole-blood data; (3) included gastric neuroendocrine tumors, gastrointestinal stromal tumors, gastric lymphoma, or other non-adenocarcinoma gastric malignancies without separately extractable GC data; (4) were reviews, editorials, letters without original patient data, conference abstracts without sufficient extractable information, case reports, or methodological articles; or (5) investigated predominantly endocrine or metabolic hormones outside the predefined neuroactive scope, including gastrin, ghrelin, secretin, glucagon-like peptide-1, glucose-dependent insulinotropic peptide, insulin, glucagon, leptin, and adiponectin, unless an eligible neuroactive mediator was simultaneously measured and separately reported.

We included untargeted or targeted metabolomics studies only when a prespecified neuroactive mediator within the scope of the review was individually identifiable and quantitative, or when we could extract direction-specific results for GC. We excluded studies in which neuroactive compounds appeared only as unreported components of a global metabolomic signature from the primary biomarker synthesis.

### 2.4. Study Selection

We exported all records retrieved from the electronic databases and consolidated them into a single dataset. Records from PubMed/MEDLINE, Scopus, and Web of Science Core Collection were harmonized using available bibliographic identifiers and citation metadata. Duplicate records were identified and removed using a hierarchical matching procedure based on PMID, DOI, and normalized article titles, with additional manual verification of ambiguous matches. We selected studies in two stages. First, we screened titles and abstracts against the predefined eligibility criteria. Second, we assessed the full texts of potentially relevant publications for final inclusion.

We retained studies that appeared potentially eligible but contained ambiguous information about the biological matrix, cancer type, analyte identity, or availability of GC-specific results for full-text assessment rather than excluding them during title/abstract screening. Full-text exclusions were documented together with the principal reason for exclusion. When multiple publications appeared to report overlapping study populations, we compared study characteristics, recruitment periods, sample sizes, and reported outcomes to determine whether they represented independent cohorts or multiple reports from the same underlying population. Complementary reports were retained when they provided nonredundant relevant information, but overlapping populations were not treated as independent studies when summarizing the evidence.

Titles and abstracts were independently screened by two reviewers against the predefined eligibility criteria. Potentially eligible reports subsequently underwent independent full-text assessment by the same reviewers. Disagreements concerning study inclusion or exclusion were resolved through discussion and consensus, with consultation of a third reviewer when necessary. Formal inter-reviewer agreement statistics, such as Cohen’s κ, were not calculated.

**Figure 1:**
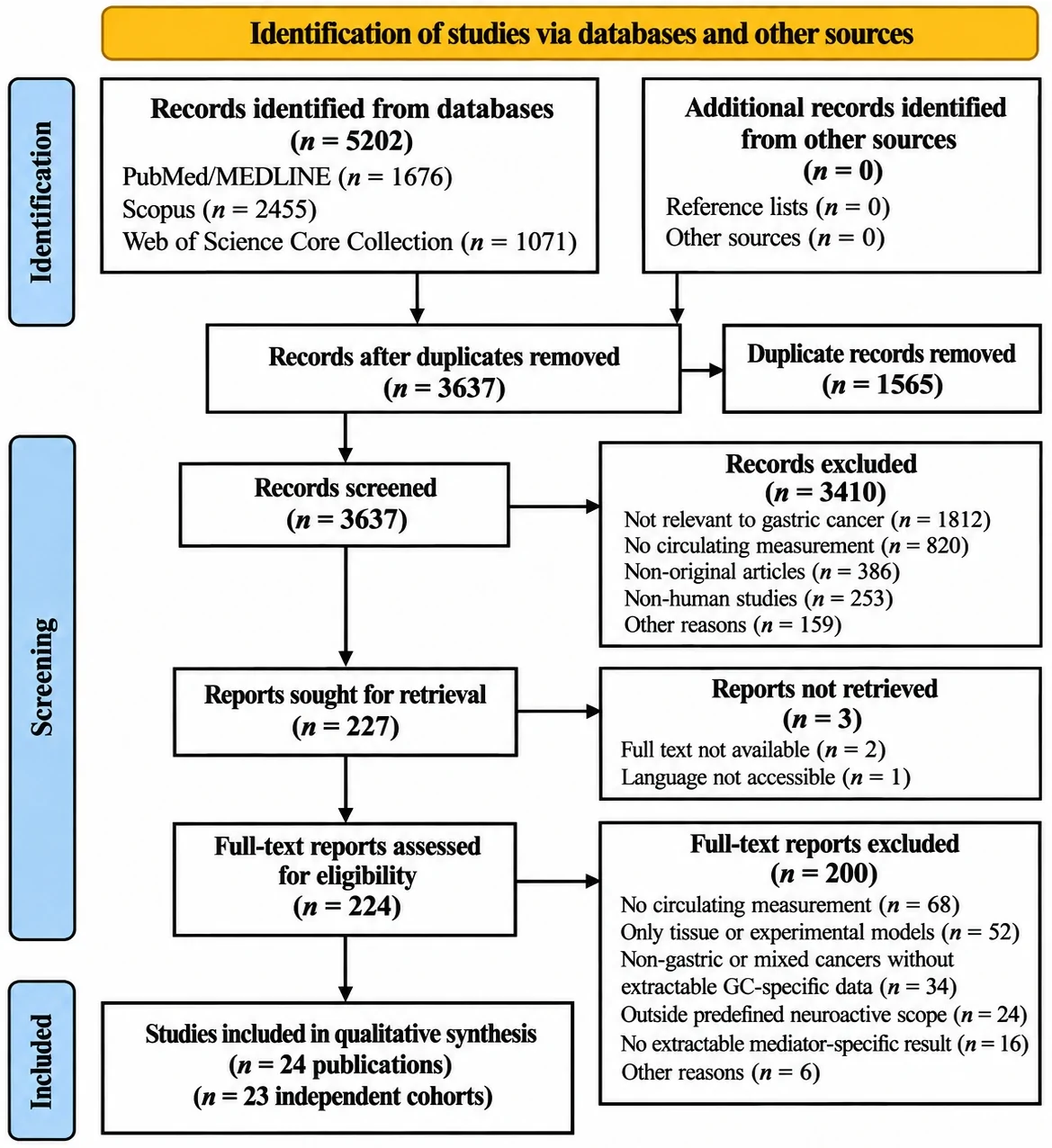
PRISMA2020 flow diagram illustrating the identification, screening, eligibility assessment, and final inclusion of studies investigating circulating neuroactive mediators in gastric cancer.

The study-selection process is summarized in a PRISMA 2020 flow diagram (Figure 1), including the numbers of records identified, duplicates removed, records screened, reports assessed in full text, exclusions with reasons, and studies included in the final structured narrative synthesis.

### 2.5. Data Extraction

Data were extracted using a predefined standardized form. For each eligible study, we recorded bibliographic information, study design, recruitment period, country, sample size, comparator groups, demographic characteristics, histological subtype, disease stage, and treatment status. We also extracted the biological matrix (serum, plasma, or whole blood), sampling and processing conditions, storage procedures, neuroactive mediators investigated, analytical methods, assay characteristics, and reported quality-control information. Missing or insufficiently documented information was classified as not reported (NR) rather than inferred.

Biomarker-related data included circulating concentrations, measures of central tendency and dispersion, between-group differences, and reported statistical significance. Where available, we extracted diagnostic-performance measures, including the area under the receiver operating characteristic curve (AUC), cutoff values, sensitivity, specificity, and confidence intervals.

Clinicopathological associations were extracted for tumor size, location, invasion depth, lymph-node involvement, distant metastasis, TNM stage, histological subtype, and tumor differentiation. For longitudinal studies, we recorded treatment type, sampling time points, and changes in circulating mediator concentrations. Prognostic outcomes included recurrence, progression, disease-free survival, progression-free survival, overall survival, hazard ratios, confidence intervals, and results from univariable or multivariable analyses, when reported.

To improve consistency and minimize extraction errors, the extracted information was checked against the corresponding original publications, with particular attention to numerical results, analytical methodologies, clinical endpoints, and the distinction between univariable and multivariable findings. Any discrepancies or ambiguities identified during verification were addressed by re-examining the source publication. Information that could not be reliably established from the published report was retained as not reported (NR), rather than reconstructed or inferred.

Data were organized at the publication, independent-cohort, and individual-biomarker levels to facilitate the structured narrative synthesis while avoiding double counting of overlapping patient populations. This organization also enabled the traceability of individual findings to their original sources and supported consistent interpretation across the different clinical evidence domains.

### 2.6. Classification of Neuroactive Mediators

To facilitate a biologically coherent synthesis, eligible and prespecified neuroactive analytes were classified into six functional groups:

(1) Classical and monoaminergic neurotransmitters, including 5-HT, DA, NE, EPI, gamma-aminobutyric acid (GABA), and targeted glutamatergic mediators where applicable;
(2) Catecholamine metabolites, including metanephrine and normetanephrine;
(3) Classical neuropeptides, including GAL, SP and related tachykinins, NPY, NT, VIP, CGRP, pituitary adenylate cyclase-activating polypeptide (PACAP), NMU, CART peptide, and somatostatin;
(4) Endogenous opioid peptides, including β-endorphin and enkephalins;
(5) The GRP pathway, including GRP and its precursor ProGRP;
(6) Selected gut–brain peptides with established neuroactive functions, including motilin, CCK, PYY, and obestatin.

We used this classification to organize the search, data extraction, and qualitative synthesis, and it was not intended to imply equivalent biochemical functions, physiological sources, or clinical relevance across analytes. We took particular care to distinguish parent neurotransmitters from their downstream metabolites and related biomarkers. Accordingly, we analyzed metanephrine and normetanephrine separately from EPI and NE, and we considered ProGRP a circulating precursor-related biomarker of the GRP pathway rather than a direct measurement of mature GRP. Similarly, measurements of serotonin obtained from whole blood, serum, and plasma were retained as distinct analytical contexts because peripheral 5-HT concentrations are strongly influenced by biological matrix and platelet contribution.

We retained prespecified mediators for which we found no eligible circulating human GC studies to distinguish an absence of eligible clinical evidence from an absence of biological or experimental relevance.

### 2.7. Risk-of-Bias and Methodological Quality Assessment

We evaluated risk of bias according to each study’s design and primary clinical purpose rather than applying a single instrument across heterogeneous study types. We assessed diagnostic-accuracy studies using the Quality Assessment of Diagnostic Accuracy Studies 2 (QUADAS-2) tool [7], considering the domains of patient selection, index test, reference standard, and flow and timing. We evaluated prognostic-factor studies using the Quality In Prognosis Studies (QUIPS) framework [8], covering study participation, study attrition, prognostic-factor measurement, outcome measurement, study confounding, and statistical analysis and reporting. We assessed observational cohort, case–control, and cross-sectional studies without a formal diagnostic-accuracy or prognostic design using the corresponding Joanna Briggs Institute (JBI) Critical Appraisal Checklists [9]. For studies addressing more than one clinical objective, we selected the appraisal tool based on the study component contributing the principal evidence to the present synthesis; we interpreted additional diagnostic, clinicopathological, longitudinal, or prognostic findings within the methodological limitations of the same study.

Because analytical reliability is particularly important for circulating neurotransmitters, neuropeptides, and related neuroactive mediators, we complemented conventional risk-of-bias assessment with a separate descriptive appraisal of analytical and pre-analytical reporting quality. The assessed items included biological matrix; fasting status; collection tube and anticoagulant; time from sample collection to processing; centrifugation conditions; storage temperature and duration, when reported; freeze–thaw exposure; assay platform; manufacturer; analytical sensitivity or limit of detection; intra- and inter-assay coefficients of variation; replicate or duplicate measurements; and reported calibration or quality-control procedures. We paid particular attention to matrix-dependent analytes, including serotonin, for which whole-blood, serum, and plasma measurements may reflect substantially different pre-analytical and biological conditions.

We maintained the analytical and pre-analytical appraisal separately from the validated risk-of-bias instruments and did not convert it into a numerical score or use it to modify QUADAS-2, QUIPS, or JBI ratings. Missing methodological information was classified as not reported rather than assumed to represent inadequate laboratory practice. These variables were instead used to contextualize between-study heterogeneity, assess the interpretability and reproducibility of circulating biomarker findings, and identify methodological limitations relevant to future validation studies.

The risk-of-bias assessment was based on the most complete source material available for each publication. For studies with accessible full texts, methodological domains were evaluated using the information reported in the original articles. For historical or non-English-language publications available only through abstracts, assessments were restricted to explicitly reported methodological details, and domains with insufficient information were classified as unclear. Publications available exclusively as bibliographic records, without sufficient information to verify eligibility or assess methodological quality, were designated as not assessable and were not used to support the qualitative evidence synthesis. Importantly, incomplete reporting was not automatically interpreted as evidence of high risk of bias. Study-specific assessments, source availability, and supporting methodological explanations are provided in Supplementary Table S5.

In addition, we performed a mediator-specific assessment of physiological and pre-analytical confounding and reporting completeness. For each mediator or mediator class, we identified the principal variables potentially affecting circulating concentrations, including platelet-related factors for serotonin; fasting status, gastrointestinal physiology, and surgical conditions for motilin, CCK, and related gut peptides; and physiological stress, medication exposure, and sampling conditions for catecholaminergic mediators. Reporting completeness was evaluated at the study level, with variables classified as reported, not reported, not applicable, or not assessable according to the available source information. Reporting frequencies were calculated using the number of assessable studies relevant to each mediator-specific variable as the denominator. Missing information was not interpreted as evidence that the corresponding confounder had been inadequately controlled. The mediator-specific assessment and reporting frequencies are presented in Supplementary Table S6.

### 2.8. Evidence Synthesis and Statistical Analysis

Given the anticipated heterogeneity in neuroactive mediators, biological matrices, analytical methods, comparator groups, disease stages, sampling contexts, and clinical endpoints, we used a structured qualitative synthesis as the primary approach. Evidence was organized by mediator or mediator family and subsequently interpreted across four partially overlapping clinical domains: diagnostic, clinicopathological, treatment-monitoring, and prognostic evidence.

Circulating concentration results were interpreted separately according to biological matrix whenever matrix-related differences could materially affect biomarker measurements. Therefore, serum, plasma, and whole-blood measurements were not assumed to be directly interchangeable. This distinction was considered particularly important for 5-HT, because platelet storage and release can substantially influence peripheral 5-HT concentrations and complicate direct comparisons among whole blood, serum, and plasma. Differences in fasting status, sample processing, storage conditions, assay platform, and sampling time relative to surgery or systemic treatment were also considered when evaluating consistency across studies.

We considered a quantitative meta-analysis a priori only when at least two independent studies evaluated the same mediator using sufficiently comparable populations, biological matrices, comparator groups, analytical approaches, and outcome definitions. We did not pool studies solely because they measured the same mediator when substantial clinical, biological, or analytical incompatibility was present. If sufficiently homogeneous data were available, we would have summarized continuous biomarker concentrations using mean differences for directly comparable measurement scales or standardized mean differences when appropriate; we would have synthesized diagnostic-accuracy and time-to-event outcomes separately using outcome-specific effect measures. We would have evaluated statistical heterogeneity using the I^2^ statistic alongside clinical and methodological heterogeneity.

After assessing the eligible studies, we did not consider quantitative pooling sufficiently justified because repeated investigations of the same mediator generally remained heterogeneous in biological matrix, analytical methodology, study population, comparator definition, sampling context, or reported outcome. Accordingly, we did not calculate an overall or mediator-specific pooled effect estimate. Results were synthesized narratively, emphasizing the direction and magnitude of reported effects where available, statistical significance, replication across independent cohorts, sample size, biological matrix, analytical methodology, clinical context, and risk of bias.

No overall pooled estimate across different neurotransmitters, neuropeptides, or related neuroactive mediators was calculated because such an effect would lack biological and clinical interpretability. Evidence from multiple reports originating from the same or substantially overlapping study population was considered at the cohort level when assessing independent replication, thereby avoiding double counting of the same underlying participants. Findings derived exclusively from non-circulating compartments were not combined with circulating biomarker results and were used only to provide mechanistic or contextual support where appropriate.

### 2.9. Translational Evidence Mapping

To distinguish clinical circulating biomarker evidence from mechanistic and biological plausibility, we mapped findings from the eligible circulating studies against selected tissue-level, receptor-level, cellular, and experimental evidence involving the same neuroactive pathways in GC. We did not include mechanistic studies in the primary systematic biomarker dataset, and they did not contribute to the number of eligible clinical studies or support independent clinical replication. Instead, we considered them separately as contextual evidence to assess whether observed circulating alterations were biologically compatible with tumor-associated neuroactive signaling and to identify pathways with substantial mechanistic evidence despite limited or absent circulating human validation.

The translational evidence map considered several complementary dimensions: the presence and direction of circulating alterations; replication across independent clinical cohorts; biological matrix and analytical context; tumor, stromal, or receptor expression; experimentally supported downstream signaling pathways; reported tumor-related phenotypes, including proliferation, invasion, migration, angiogenesis, treatment resistance, or other relevant cancer-associated processes; and major gaps between mechanistic evidence and clinical biomarker validation. Circulating alterations were not assumed to originate directly from the tumor, because measured concentrations may also reflect neural, enteroendocrine, platelet, immune, metabolic, or other systemic sources.

**Figure 2:**
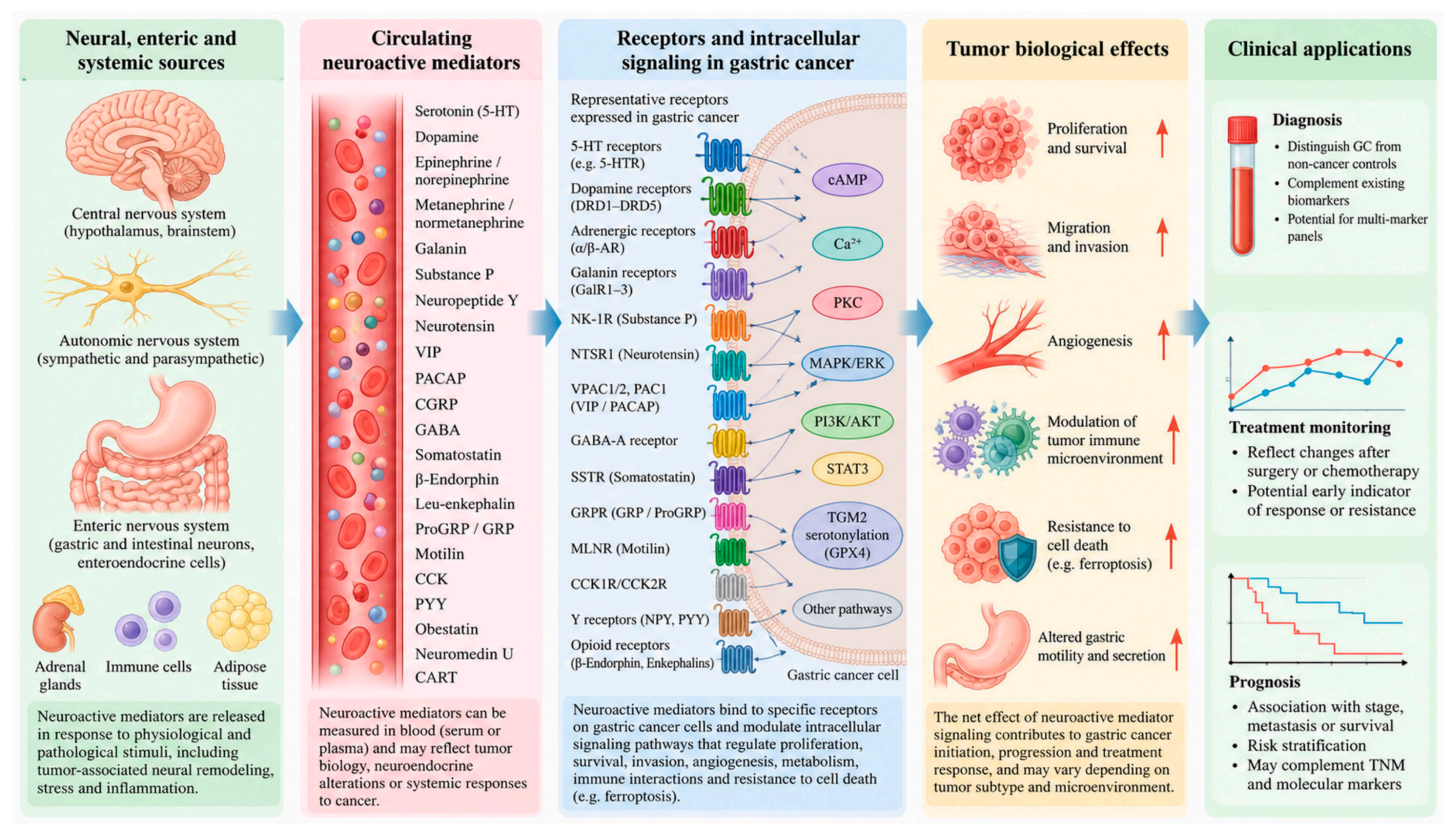
From neural and enteric signaling to circulating neuroactive biomarkers in gastric cancer: an integrated translational framework. Graphical illustration generated with the assistance of ChatGPT (OpenAI, GPT-5.5) based on detailed author-designed scientific concepts and subsequently reviewed and approved by the authors. The figure illustrates the proposed continuum linking neural, enteric, and systemic sources of neuroactive mediators with circulating biomarker alterations, receptor-mediated signaling in gastric cancer (GC), tumor-associated biological effects, and potential clinical applications. Neurotransmitters, neuropeptides, and related neuroactive mediators may originate from the central and autonomic nervous systems, enteric neurons and enteroendocrine cells, adrenal glands, immune cells, adipose tissue, and, potentially, tumor-associated neural and cellular compartments. Once released into the circulation, these mediators can be quantified in serum or plasma and may reflect tumor-associated neuroendocrine remodeling, systemic stress responses, inflammation, or changes in gastrointestinal physiology. At the tumor level, neuroactive mediators may interact with specific receptors expressed by GC cells and components of the tumor microenvironment, including serotonergic, dopaminergic, adrenergic, galanin, neurokinin-1, neurotensin, VIP/PACAP, GABAergic, somatostatin, GRP, motilin, CCK, NPY/PYY, and opioid receptor systems. Receptor activation can modulate intracellular signaling pathways such as cAMP, Ca^2+^-dependent signaling, PKC, MAPK/ERK, PI3K/AKT, and STAT3. Serotonin may also influence tumor biology through TGM2-mediated serotonylation and GPX4 stabilization, thereby contributing to ferroptosis resistance. Collectively, these pathways may affect tumor-cell proliferation and survival, migration and invasion, angiogenesis, immune–tumor interactions, resistance to cell death, and gastric motility or secretion. *Symbols:* The large horizontal blue arrows indicate the conceptual progression from the biological sources of neuroactive mediators to their appearance in the circulation, subsequent interaction with tumor-associated receptors and intracellular pathways, resulting biological effects, and potential clinical applications. The smaller arrows within the signaling panel indicate receptor-to-intracellular-pathway relationships and represent mechanistically plausible or experimentally supported signaling connections, not evidence that every circulating mediator activates every depicted pathway in gastric cancer. The upward red arrows in the tumor-effects panel indicate promotion or enhancement of the corresponding tumor-associated biological process; they do not represent measured changes in circulating mediator concentrations. Clinically, circulating neuroactive mediators may contribute to GC diagnosis, complement established biomarkers, monitor changes after surgery or systemic therapy, and provide prognostic information related to tumor burden, stage, metastasis, or survival. These applications remain at different levels of evidence and should therefore be interpreted as a translational framework rather than as established clinical indications. *Abbreviations:* 5-HT, 5-hydroxytryptamine; AKT, protein kinase B; AR, adrenergic receptor; cAMP, cyclic adenosine monophosphate; CART, cocaine- and amphetamine-regulated transcript; CCK, cholecystokinin; CCKR, cholecystokinin receptor; CGRP, calcitonin gene-related peptide; DRD, dopamine receptor; GABA, gamma-aminobutyric acid; GAL, galanin; GalR, galanin receptor; GC, gastric cancer; GPX4, glutathione peroxidase 4; GRP, gastrin-releasing peptide; GRPR, gastrin-releasing peptide receptor; MAPK/ERK, mitogen-activated protein kinase/extracellular signal-regulated kinase; MLNR, motilin receptor; NK-1R, neurokinin-1 receptor; NPY, neuropeptide Y; NTSR1, neurotensin receptor 1; PAC1, pituitary adenylate cyclase-activating polypeptide type I receptor; PACAP, pituitary adenylate cyclase-activating polypeptide; PI3K, phosphoinositide 3-kinase; PKC, protein kinase C; ProGRP, progastrin-releasing peptide; PYY, peptide YY; SSTR, somatostatin receptor; STAT3, signal transducer and activator of transcription 3; TGM2, transglutaminase 2; VIP, vasoactive intestinal peptide; VPAC, vasoactive intestinal peptide receptor.

Particular attention was given to discrepancies between the strength of mechanistic evidence and the maturity of circulating biomarker evidence. Neuroactive pathways supported predominantly by tissue, receptor-expression, cellular, or experimental studies but lacking eligible circulating human GC data were classified as mechanistically supported but clinically unvalidated in the circulating compartment. Conversely, circulating associations with limited mechanistic characterization were interpreted as exploratory biomarker signals rather than evidence of established tumor-driving pathways. This framework was used to identify replicated signals, analytically heterogeneous findings, and translational evidence gaps and forms the basis of the synthesis summarized in Supplementary Table S3 and the conceptual model presented in Figure 2.

Taken together, the evidence summarized in Supplementary Table S3 highlights a marked imbalance between the biological plausibility of neuroactive signaling in GC and the maturity of circulating biomarker validation. While several mediators have been investigated in human blood, only a limited subset has been examined across independent clinical cohorts, and circulating concentrations may not originate directly from the tumor. Rather, they may integrate signals derived from the central and autonomic nervous systems, the enteric neuroendocrine compartment, immune and metabolic tissues, and the tumor microenvironment. To place these heterogeneous observations within a unified translational framework, Figure 2 illustrates the proposed continuum from neural, enteric, and systemic sources of circulating neuroactive mediators to receptor-mediated signaling in GC cells, downstream tumor-biological effects, and potential diagnostic, treatment-monitoring, and prognostic applications.

### 2.10. Use of Generative Artificial Intelligence

Generative artificial intelligence (GenAI), specifically ChatGPT (OpenAI, GPT-5.5), was used exclusively to assist in preparing the graphical illustrations included in this review (Figure 2). The authors conceived the scientific concepts, biological mechanisms, figure organization, and visual content of each figure and provided detailed iterative instructions to generate graphical renderings that accurately reflected the evidence synthesized in the manuscript. The AI tool was used solely for visual illustration and graphical rendering and was not used for literature searching, study selection, evidence synthesis, data extraction, data analysis, interpretation of findings, or manuscript writing. The authors critically reviewed, verified, and approved all graphical outputs and took full responsibility for the scientific accuracy, integrity, and final content of the figures and the manuscript.

## 3. Results

### 3.1. Study Selection and Characteristics of Included Studies

The systematic search identified 5202 records, including 1676 from PubMed/MEDLINE, 2455 from Scopus, and 1071 from Web of Science Core Collection. After removal of 1565 duplicates, 3637 unique records underwent title and abstract screening. Of these, 3410 were excluded, and 227 reports were sought for retrieval. Three reports could not be retrieved, leaving 224 full-text reports for eligibility assessment. Following the exclusion of 200 reports, 24 publications representing 23 independent cohorts were included in the final qualitative synthesis (Figure 1).

The included studies were predominantly observational and exploratory, with considerable variation in comparator populations, biological matrices, analytical methods, and clinical endpoints. Most investigated diagnostic or case–control differences and clinicopathological associations, whereas longitudinal treatment-monitoring and prognostic evaluations were less frequent. These differences limited direct comparisons and the identification of consistently replicated biomarker signals.

The evidence encompassed monoaminergic neurotransmitters, catecholamine metabolites, classical neuropeptides, endogenous opioid peptides, and gut–brain mediators. Although serotonin, galanin, motilin, cholecystokinin, peptide YY/obestatin, and catecholamine-related metabolites were investigated in multiple reports, independent replication remained limited for most analytes. No eligible circulating human GC evidence was identified for several experimentally relevant mediators, including GABA, CGRP, PACAP, NMU, and CART peptide.

An important source of heterogeneity was the transition from historical blood-based investigations to contemporary serum and plasma assays, including targeted mass spectrometry and metabolomic approaches. Differences in biological matrix, sample processing, and analytical reporting were particularly relevant for serotonin, whose peripheral concentrations are strongly influenced by platelet-related factors. Consequently, apparent differences in biomarker direction across studies cannot be interpreted independently of their analytical context.

Overall, the available literature provides a heterogeneous and incompletely replicated evidence base, with substantially greater emphasis on diagnostic and clinicopathological associations than on treatment monitoring or survival.

A comprehensive overview of the 24 eligible publications, including study characteristics, circulating biomarker findings, diagnostic performance, clinicopathological associations, treatment-associated changes, and prognostic evidence, is provided in Supplementary Table S4.

### 3.2. Circulating Neurotransmitters and Monoaminergic Mediators

Circulating evidence for classical neurotransmitters in GC remains limited and methodologically heterogeneous. The available human studies have primarily investigated 5-HT, DA, and metabolites of the catecholaminergic pathway. No eligible studies directly quantifying circulating NE or EPI in patients with GC were identified. Differences in biological matrices, analytical platforms, and pre-analytical procedures limit the comparability of circulating concentrations and complicate the interpretation of potentially divergent findings, particularly for serotonin.

#### 3.2.1. Serotonin (5-HT)

5-HT is among the earliest and most frequently investigated circulating neuroactive mediators in GC. Historical whole-blood investigations generally suggested reduced serotonin concentrations in patients with GC, although incomplete analytical reporting and limited control of pre-analytical variables restrict the interpretation of these findings [10,11,12].

Contemporary investigations have yielded a more complex picture. Targeted analysis of serum tryptophan metabolites identified alterations in tryptophan metabolism, particularly involving the kynurenine pathway, without establishing circulating 5-HT as an independently validated diagnostic marker [13]. In contrast, plasma metabolomic evidence demonstrated increased circulating 5-HT in GC [5]. This observation was supported by experimental findings linking 5-HT to TGM2-mediated serotonylation and stabilization of GPX4, suppression of ferroptosis, and promotion of gastric tumorigenesis [5].

The apparent divergence between historical reductions in blood 5-HT and contemporary increases in plasma 5-HT highlights the importance of biological matrix and analytical context rather than necessarily indicating conflicting biological effects. Because peripheral serotonin is predominantly stored in platelets, whole blood, serum, and platelet-poor plasma represent distinct analytical compartments with different susceptibility to platelet-related interference. Variability in sample processing, analytical methodology, and patient characteristics further limits direct comparisons.

Overall, circulating 5-HT represents a biologically relevant but analytically challenging biomarker candidate. Its potential clinical utility requires prospective validation using standardized sample collection, platelet-controlled measurements, and clearly defined clinical endpoints.

#### 3.2.2. Dopamine (DA)

Clinical evidence concerning circulating DA in GC remains limited to a single investigation reporting substantially reduced plasma concentrations in patients with GC compared with healthy controls [14]. Complementary analyses of DA receptor expression and catechol-O-methyltransferase (COMT) in peripheral blood mononuclear cells and gastric tissues suggested broader alterations in dopaminergic signaling, although these tissue- and cell-associated findings cannot independently establish the diagnostic relevance of circulating DA [14].

Despite this biologically relevant observation, researchers have not independently replicated the circulating finding, and validated diagnostic thresholds, longitudinal treatment-related changes, and prognostic associations remain unavailable. Consequently, plasma DA should currently be regarded as an exploratory biomarker candidate requiring prospective validation in larger cohorts with standardized analytical procedures.

#### 3.2.3. Catecholamine Metabolites

Researchers have investigated circulating metanephrine and normetanephrine as indirect indicators of catecholaminergic activity in GC. Importantly, these metabolites are biochemically distinct from their parent catecholamines, EPI and NE, and their measurement should not be interpreted as direct quantification of circulating catecholamines [15].

The available evidence ranges from a small exploratory investigation reporting preliminary clinicopathological associations to a larger clinical study demonstrating relationships between circulating catecholamine metabolites and tumor aggressiveness [15,16]. In the latter study, elevated metanephrine was associated with greater invasion depth, nodal involvement, and advanced TNM stage, whereas normetanephrine was also associated with larger tumor size [15].

Prognostic findings further differentiated the two metabolites. Higher normetanephrine concentrations were associated with poorer two-year survival (HR = 2.289; log-rank *p* = 0.0104), whereas the corresponding association for metanephrine was not statistically significant (HR = 1.462; log-rank *p* = 0.1487) [15]. Because these findings were derived from univariable survival analyses, they support an exploratory prognostic association rather than an independently validated prognostic effect.

Overall, circulating catecholamine metabolites may reflect tumor burden, with normetanephrine showing an additional potential prognostic signal. However, sympathetic activation, stress, medications, posture, and sampling conditions influence their concentrations. Independent validation, standardized pre-analytical protocols, and multivariable adjustment for relevant confounders are therefore required before establishing clinical applicability.

#### 3.2.4. Overall Evidence for Monoaminergic Circulating Markers

Overall, circulating monoaminergic markers in GC show biologically relevant but insufficiently replicated alterations, with substantial differences in analytical reliability and clinical evidence across mediator classes. Historical and contemporary serotonin findings remain difficult to reconcile because of matrix-dependent measurements and pre-analytical variability [5,10,11,12]. Plasma dopamine shows a potentially relevant alteration, but evidence remains limited to a single clinical investigation [14]. Among catecholamine metabolites, normetanephrine provides the clearest preliminary prognostic signal, although its association with survival has not been established as independent of clinicopathological confounders [15].

Important translational gaps persist. No eligible circulating human GC studies directly quantified NE, EPI, or GABA, despite their established relevance in experimental investigations of neuroactive signaling. This discrepancy underscores the need to distinguish mechanistic plausibility from clinically validated evidence from circulating biomarkers.

Taken together, these findings support further investigation of monoaminergic pathways using standardized analytical procedures, independent clinical cohorts, and clearly defined diagnostic and prognostic endpoints. The following section examines the broader evidence concerning circulating neuropeptides and gut–brain mediators.

### 3.3. Circulating Neuropeptides and Related Neuroactive Peptides

Compared with classical neurotransmitters, researchers have investigated a broader range of circulating neuropeptides and related mediators in GC, although the evidence remains heterogeneous and unevenly replicated. Several mediators, including GAL, motilin, CCK, PYY, and obestatin, have been evaluated in multiple reports, whereas evidence for SP, NPY, NT, VIP, somatostatin, endogenous opioid peptides, and ProGRP remains more limited. Importantly, multiple publications do not necessarily indicate independent validation, particularly when patient populations overlap or measurements are restricted to perioperative settings.

#### 3.3.1. Galanin (GAL)

GAL exhibited one of the most consistent circulating patterns among the neuropeptides investigated in GC. Two independent clinical cohorts reported significantly lower circulating GAL concentrations in patients with GC than in healthy controls, despite differences in biological matrix [17,18].

Beyond the case–control difference, lower plasma GAL concentrations were associated with greater tumor size, lymph-node involvement, and more advanced TNM stage. A postoperative increase toward control concentrations further suggested a relationship between circulating GAL and tumor presence [17]. Complementary alterations in tumor-associated GAL/GalR expression provided biological context but did not independently support circulating biomarker performance.

The concordant findings across independent cohorts, together with clinicopathological and postoperative associations, position GAL among the more promising circulating neuropeptide candidates. Nevertheless, available studies remain small, and they lack externally validated diagnostic thresholds, longitudinal recurrence-monitoring data, and survival associations.

#### 3.3.2. Substance P (SP) and Endogenous Opioid Peptides

A historical clinical investigation found that circulating SP and β-endorphin were significantly elevated in patients with advanced GC compared with healthy controls [6]. However, these findings were not accompanied by comparable evidence for all peptides investigated. In particular, Leu-enkephalin showed no convincing change in serum, despite changes in gastric juice, underscoring the distinction between systemic and local gastrointestinal peptide responses [19].

Although increased SP and β-endorphin may reflect neuroimmune or neuroendocrine alterations associated with advanced malignancy, the evidence derives from a single cohort and lacks independent validation. Moreover, pain, physiological stress, medication exposure, and nutritional disturbances may influence circulating peptide concentrations. Consequently, neither SP nor the investigated endogenous opioid peptides are currently validated circulating biomarkers of GC.

#### 3.3.3. Neuropeptide Y (NPY)

Available clinical evidence suggests an inverse relationship between circulating NPY and selected indicators of disease burden in GC. Lower plasma NPY concentrations were associated with more advanced disease, larger tumors, and greater body-weight loss, whereas no significant associations were identified with invasion depth, histological differentiation, or lymph-node involvement [20].

Importantly, the reported reduction in plasma NPY relative to healthy controls was calculated for a combined gastric and colorectal cancer population rather than a GC-specific cohort. Accordingly, this finding cannot establish a GC-specific diagnostic difference, although the separately reported clinicopathological associations remain relevant to the present review [20].

These preliminary observations suggest a possible relationship between circulating NPY and tumor-associated or systemic metabolic alterations. However, the absence of independent GC-specific validation and the potential influence of nutritional status and disease-related weight loss preclude conclusions regarding its diagnostic or prognostic utility.

#### 3.3.4. Neurotensin and Vasoactive Intestinal Peptide

Circulating evidence for NT and VIP remains limited and does not establish a consistently replicated GC-specific biomarker profile. Multiparametric neuropeptide investigations reported heterogeneous circulating patterns, while earlier VIP-related findings were more prominent in gastric juice than in serum [19,21].

In contrast, researchers have reported increased plasma NT in GC, along with a positive association with MMP-9 activity [22]. Complementary experimental studies linked NT/NTSR1 signaling to MMP-9 activation, gastric cancer cell migration, and invasion through PKC-, ERK-, and PI3K-related pathways [23,24]. Although these findings provide mechanistic support for NT involvement in GC progression, they do not independently replicate its circulating alteration in clinical settings.

Thus, NT represents a biologically plausible but clinically unvalidated circulating candidate, whereas the available evidence remains insufficient to define a reproducible circulating VIP phenotype in GC.

#### 3.3.5. Cholecystokinin

Evidence on circulating CCK in GC comes from two distinct clinical contexts: perioperative investigations of gastrointestinal physiology and case–control evaluations of biomarker performance. Early studies documented changes in CCK release after gastrectomy, but overlapping clinical populations and experimental settings prevent interpretation as independent replications [25,26].

More recent case–control investigations reported lower plasma CCK concentrations in patients with GC than in healthy controls, with one study additionally demonstrating promising diagnostic discrimination [21,27]. However, these findings should be distinguished from perioperative physiological responses, which may reflect altered gastrointestinal anatomy and function rather than tumor-specific changes.

Although the concordant direction of the case–control findings supports further investigation of CCK as a circulating candidate, its sensitivity to food intake, gastric emptying, vagal signaling, and postoperative physiology necessitates standardized sampling and independent diagnostic validation.

#### 3.3.6. Peptide YY (PYY) and Obestatin

Researchers have investigated circulating PYY and obestatin in both case–control and perioperative settings, but the substantial physiological consequences of gastric resection complicate their interpretation. For PYY, available cross-sectional evidence has not established a reproducible GC-specific alteration, whereas longitudinal investigations demonstrated postoperative changes in PYY3-36 that were influenced, at least in part, by vagal nerve preservation and altered gastrointestinal physiology [21,28,29].

Obestatin showed a different pattern, with one case–control study reporting markedly elevated serum concentrations in GC and an inverse association with circulating GAL [18]. Additional perioperative investigations documented changes in obestatin following gastrectomy, although these responses cannot independently confirm the tumor specificity of the case–control finding [29,30].

Overall, PYY and obestatin appear particularly sensitive to changes in gastrointestinal anatomy and neurophysiology. While increased circulating obestatin represents a preliminary GC-associated signal, postoperative fluctuations in either peptide should not be interpreted as evidence of tumor-related biomarker dynamics without accounting for surgical and physiological confounders.

#### 3.3.7. Somatostatin

Historical clinical evidence indicated increased somatostatin-like immunoreactivity in peripheral blood from patients with GC compared with healthy individuals and patients with benign gastroduodenal disease [31]. Although complementary gastric venous measurements were obtained, these findings represent a distinct sampling compartment and should not be conflated with peripheral circulating concentrations.

The reported elevation remains an isolated observation without independent confirmation using contemporary analytical methods. Consequently, circulating somatostatin is not currently a validated diagnostic or prognostic biomarker in GC.

#### 3.3.8. Motilin

Motilin is among the few circulating gut–brain peptides that show a concordant direction of change across independent GC investigations. Two clinical studies reported higher plasma motilin concentrations than in healthy controls [24,32].

However, longitudinal observations revealed substantial perioperative variability, with a marked decline immediately after gastrectomy followed by a return toward preoperative concentrations within the first postoperative week [32]. The authors also reported associations with disease stage, although the available findings do not establish a consistent relationship between motilin and tumor progression.

These observations suggest that circulating motilin reflects both GC-associated alterations and changes in gastrointestinal physiology. Its sensitivity to fasting, gastric motility, nutritional intake, and surgical intervention limits its specificity to tumors and requires standardized sampling before diagnostic or monitoring applications can be evaluated.

#### 3.3.9. Progastrin-Releasing Peptide (ProGRP)

ProGRP has undergone comparatively extensive evaluation within a single clinical GC cohort, encompassing case–control differences, clinicopathological associations, diagnostic performance, and treatment-related changes [33]. Serum concentrations were substantially higher in patients with GC than in individuals with benign gastric disease or healthy controls and were positively associated with tumor size, invasion depth, nodal involvement, advanced TNM stage, and poorer differentiation. They also showed positive correlations with CEA and CA72-4 [33].

Diagnostic analysis yielded an AUC of 0.713 (95% CI: 0.631–0.795), with reported sensitivity and specificity of 85.9% and 81.2%, respectively, at a ProGRP cutoff of 28.3 pg/mL. This threshold was derived from ROC analysis of the original study data rather than being prespecified. However, the exact criterion used for cutoff optimization and the composition of the reference population used to estimate diagnostic performance were not explicitly described. Combining ProGRP with CEA and CA72-4 increased the reported sensitivity and specificity to 91.2% and 93.4%, respectively [33]. Nevertheless, these findings originated from a single study without reported internal validation or independent external validation. Consequently, the diagnostic estimates should be interpreted as preliminary, potentially optimistic performance estimates, particularly because data-driven threshold selection may inflate sensitivity and specificity.

A longitudinal treatment component also showed a substantial decline in serum ProGRP after chemotherapy in patients with advanced GC [33]. Although this finding suggests potential utility for treatment monitoring, the reported baseline and follow-up concentrations should not be interpreted as a fully characterized paired subgroup analysis without further methodological clarification.

Overall, ProGRP represents a potentially informative circulating candidate, but its diagnostic and monitoring performance requires independent prospective validation. Importantly, the measured circulating analyte was ProGRP rather than mature GRP; therefore, evidence on GRP/GRPR signaling should be considered mechanistic context rather than direct evidence of circulating GRP.

#### 3.3.10. Overall Pattern of Circulating Neuropeptide Evidence

The circulating neuropeptide literature reveals distinct levels of clinical evidence rather than a uniform GC-associated biomarker profile. GAL and motilin show the most consistent directional findings across independent cohorts, although their clinical interpretation differs: reduced GAL has been associated with tumor burden, whereas increased motilin is strongly influenced by gastrointestinal physiology and perioperative changes [17,18,24,32]. CCK, PYY, and obestatin have also been investigated repeatedly, but overlapping cohorts, heterogeneous clinical settings, and surgery-related physiological effects limit the interpretation of these findings as independent biomarker validation [18,21,25,26,27,28,29,30].

ProGRP provides the most comprehensive single-cohort evidence regarding diagnostic discrimination, clinicopathological associations, and treatment-related changes, but external validation remains unavailable [33]. NT combines a preliminary circulating signal with mechanistic evidence involving NT/NTSR1–MMP-9 signaling, whereas the circulating evidence for SP, β-endorphin, NPY, somatostatin, VIP, and Leu-enkephalin remains limited or unreplicated [19,20,22,23,31].

We identified no eligible circulating human GC studies for several experimentally relevant neuropeptides, including CGRP, PACAP, NMU, and CART peptide. This gap highlights the difference between mechanistic involvement in tumor biology and clinically validated evidence for circulating biomarkers.

### 3.4. Diagnostic Performance of Circulating Neuroactive Mediators

Formal diagnostic-accuracy assessments were available for only a minority of circulating neuroactive mediators investigated in GC. Most studies reported case–control concentration differences without ROC analysis, validated thresholds, or independent testing. Consequently, statistical differences between groups should not be interpreted as evidence of clinically established diagnostic performance, particularly when comparator populations consisted predominantly of healthy individuals rather than patients with benign or premalignant gastric conditions.

#### 3.4.1. ProGRP

ProGRP was among the few mediators evaluated using formal ROC analysis. The reported AUC was 0.713 (95% CI 0.631–0.795), with sensitivity and specificity of 85.9% and 81.2%, respectively, at a cutoff of 28.3 pg/mL [33]. Combining ProGRP with CEA and CA72-4 yielded higher reported sensitivity (91.2%) and specificity (93.4%), suggesting potential complementary diagnostic value within a multimarker approach. Although the investigation included both healthy controls and patients with benign gastric disease, all diagnostic estimates originated from a single cohort without external validation or a prospectively established threshold. Accordingly, these findings support preliminary diagnostic potential rather than clinically validated accuracy. Importantly, the reported diagnostic performance was based on a data-derived ProGRP cutoff of 28.3 pg/mL rather than a prospectively prespecified threshold. Although the original study included patients with gastric cancer, benign gastric diseases, and healthy controls, the exact composition of the reference population used for ROC analysis was not explicitly clarified. Furthermore, neither the cutoff optimization criterion nor internal validation procedures were reported, and independent external validation was unavailable. These methodological limitations raise concerns regarding potential optimism in the reported sensitivity and specificity, particularly when threshold selection and performance estimation are performed using the same dataset. Accordingly, the diagnostic utility of ProGRP, either alone or in combination with conventional tumor markers, remains exploratory and requires confirmation in independent, clinically representative cohorts using prespecified or independently validated diagnostic thresholds.

#### 3.4.2. Cholecystokinin

CCK has shown concordantly reduced circulating concentrations in GC across independent case–control investigations, with one study additionally assessing diagnostic discrimination [21,27]. However, earlier perioperative studies primarily addressed CCK physiology after gastrectomy and should not be interpreted as evidence of diagnostic accuracy or independent biomarker validation [28,29].

Despite these preliminary findings, diagnostic performance remains insufficiently validated in prospective cohorts incorporating benign and premalignant gastric conditions. Moreover, circulating CCK’s strong dependence on feeding status and gastrointestinal physiology underscores the need for standardized sampling before establishing its clinical diagnostic utility.

#### 3.4.3. Galanin

GAL showed reproducible case–control differences across two independent cohorts, with consistently lower circulating concentrations in patients with GC [17,18]. Although the concordant findings and postoperative increase support a relationship with tumor presence, neither study established externally validated diagnostic thresholds or formal performance in clinically relevant gastric disease populations [17].

Thus, GAL represents a replicated circulating alteration with potential diagnostic relevance, but its ability to distinguish GC from benign or premalignant gastric conditions remains undetermined.

#### 3.4.4. Obestatin

A marked increase in circulating obestatin has been reported in GC, suggesting potential case–control discrimination [18]. However, subsequent perioperative investigations primarily demonstrated sensitivity to gastric resection and postoperative physiology rather than independent replication of diagnostic performance [29,30].

The absence of validated thresholds, independent diagnostic cohorts, and comparisons with benign or premalignant gastric disease limits obestatin to an exploratory circulating candidate.

#### 3.4.5. Dopamine and Catecholamine Metabolites

Although GC has been associated with substantially reduced plasma DA concentrations, available investigations have not established independently validated diagnostic thresholds or reproducible ROC-based performance [14].

For metanephrine and normetanephrine, the principal findings concern clinicopathological associations and exploratory survival outcomes rather than diagnostic classification [15,16]. Their sensitivity to sympathetic activation and non-malignant physiological influences further complicates diagnostic interpretation. Consequently, none of these catecholaminergic markers currently have sufficient evidence for clinical diagnostic application in GC.

#### 3.4.6. Other Neuropeptides and Neuroactive Mediators

Several additional mediators have shown circulating alterations in GC, but diagnostic validation remains insufficient. These include SP, β-endorphin, somatostatin, and motilin, whereas the available NPY case–control comparison involved a mixed gastric and colorectal cancer population and therefore does not establish GC-specific diagnostic discrimination [19,20,24,31,32].

NT represents a distinct candidate because increased circulating concentrations were accompanied by diagnostic assessment and complementary mechanistic evidence. Nevertheless, its diagnostic performance remains externally unvalidated, and subsequent NTSR1 investigations do not independently replicate it as a circulating biomarker [22,23].

For serotonin, opposing directions reported in historical blood studies and contemporary plasma metabolomics, together with substantial matrix-dependent variability, preclude deriving a common diagnostic threshold [5,10,11,12]. Evidence for circulating VIP, PYY, and Leu-enkephalin likewise remains insufficient to establish reliable GC-specific diagnostic discrimination.

Accordingly, these findings should be interpreted as exploratory circulating signals rather than evidence of clinically validated diagnostic accuracy.

#### 3.4.7. Overall Diagnostic Evidence

Overall, the evidence reveals a substantial gap between statistically significant circulating alterations and clinically validated diagnostic performance. Although several neuroactive mediators have demonstrated case–control differences, formal diagnostic assessment remains uncommon, and independent prospective validation is lacking.

ProGRP has one of the most developed diagnostic datasets, including ROC analysis, comparisons with benign gastric disease, and multimarker evaluation [33]. CCK has shown concordant reductions across clinical investigations, with dedicated diagnostic assessment also reported [21,27]. GAL shows independently replicated case–control differences, whereas obestatin and DA have more limited diagnostic evidence [14,17,18].

A major limitation is the predominance of healthy-control comparisons, which may overestimate diagnostic performance in symptomatic populations. Furthermore, the incremental value of neuroactive mediators beyond established tumor markers and routine clinical assessment remains largely unexplored.

Consequently, no circulating neuroactive mediator can currently be recommended as a validated stand-alone diagnostic biomarker for GC. Future studies should prioritize prospective recruitment, clinically relevant benign and premalignant comparators, standardized sampling, prespecified diagnostic thresholds, and independent external validation, including assessment of incremental diagnostic value.

### 3.5. Clinicopathological Associations of Circulating Neuroactive Mediators

Several circulating neuroactive mediators have been associated with tumor burden, disease stage, and other clinicopathological characteristics of GC, particularly GAL, ProGRP, NPY, motilin, metanephrine, and normetanephrine. However, these associations were heterogeneous, often derived from single cohorts, and rarely independently validated.

#### 3.5.1. Galanin

Lower circulating GAL concentrations were associated with greater tumor burden, including larger tumor size, lymph-node involvement, and more advanced TNM stage [17]. However, no significant associations were identified with invasion depth or histological differentiation.

Although an independent cohort confirmed reduced circulating GAL in GC, the detailed clinicopathological associations were not independently replicated [18]. Thus, GAL may reflect selected aspects of disease burden, but its clinical value as a circulating marker of tumor progression remains unvalidated.

#### 3.5.2. Neuropeptide Y

Lower plasma NPY concentrations were associated with more advanced GC stage, larger tumors, and greater body-weight loss, whereas no significant relationships were identified with invasion depth, histological differentiation, or lymph-node involvement [20].

Given the involvement of NPY in appetite regulation and energy homeostasis, these associations may reflect both tumor-related changes and systemic nutritional or metabolic disturbances. Importantly, the GC-specific clinicopathological findings should be distinguished from the pooled gastric and colorectal cancer case–control comparison reported in the same study.

In the absence of independent GC-specific validation, circulating NPY remains an exploratory marker of disease burden rather than an established indicator of tumor progression.

#### 3.5.3. Motilin

Available clinicopathological evidence for circulating motilin is limited to stage-related variation reported in one cohort, with higher concentrations observed in stage II than in stage III GC [32]. This finding does not support a consistent increase in motilin with advancing disease.

Although elevated circulating motilin has been independently reported in GC [24,32], its substantial dependence on gastrointestinal motility, fasting status, nutritional intake, and perioperative physiology complicates interpretation of stage-related differences. Consequently, current evidence does not establish motilin as a reliable circulating indicator of tumor burden or disease progression.

#### 3.5.4. Progastrin-Releasing Peptide (ProGRP)

ProGRP demonstrated the broadest range of reported clinicopathological associations, with higher circulating concentrations linked to larger tumors, greater invasion depth, nodal involvement, poorer histological differentiation, and advanced TNM stage [33]. Positive correlations with CEA and CA72-4 were also observed, suggesting potential overlap with established markers of tumor burden.

However, the study did not establish whether ProGRP provided independent clinicopathological information after adjustment for conventional tumor markers and other clinical variables. Moreover, all reported associations originated from a single cohort without independent replication. Thus, ProGRP remains a promising but unvalidated circulating indicator of disease burden.

#### 3.5.5. Metanephrine and Normetanephrine

Circulating metanephrine and normetanephrine were associated with several indicators of advanced GC in a dedicated clinical cohort [15]. Higher metanephrine concentrations were linked to greater invasion depth (T3–T4), more extensive nodal involvement (N ≥ 2), and advanced TNM stage, whereas normetanephrine additionally showed an association with larger tumor diameter.

Both metabolites also exhibited variation across selected histological and tumor-location categories, including exploratory differences involving signet-ring cell carcinoma. However, these subgroup findings were heterogeneous and require independent confirmation before subtype-specific conclusions can be drawn [15].

Although the observed associations suggest a relationship with disease severity, circulating metanephrine and normetanephrine are susceptible to sympathoadrenal activation, physiological stress, comorbidities, medication exposure, and pre-analytical influences. Consequently, their clinicopathological associations cannot be interpreted as evidence of tumor-specific catecholamine production or signaling.

#### 3.5.6. Neurotensin and Other Neuroactive Mediators

Clinicopathological evidence remains limited for most other circulating neuroactive mediators. NT represents a notable example because increased circulating concentrations were associated with MMP-9 activity, while complementary experimental studies implicated NT/NTSR1 signaling in gastric cancer cell migration and invasion [22,23]. However, these mechanistic findings do not establish independently validated associations between circulating NT and individual TNM components, tumor size, histological grade, or metastatic status.

For serotonin, somatostatin, SP, β-endorphin, VIP, and Leu-enkephalin, available clinical investigations primarily addressed circulating concentration differences rather than systematic clinicopathological relationships [10,11,12,19,31]. Similarly, studies of CCK, PYY, and obestatin provided insufficient independent evidence concerning their associations with tumor stage or histopathological characteristics [18,21,27,28,29,30].

Although reduced circulating DA has been accompanied by complementary investigations of dopaminergic signaling, reproducible associations between plasma DA and specific clinicopathological characteristics have not been established [14]. Overall, the available evidence does not support definitive conclusions regarding the utility of these mediators as circulating indicators of GC aggressiveness.

#### 3.5.7. Overall Clinicopathological Evidence

Overall, circulating neuroactive alterations were most frequently associated with tumor burden and disease stage, although the strength and direction of these relationships varied across mediators. Higher ProGRP, metanephrine, and normetanephrine concentrations and lower GAL and NPY concentrations were associated with selected indicators of more advanced GC [15,17,20,33]. Motilin showed less consistent stage-related variation, potentially reflecting its substantial dependence on gastrointestinal physiology [32].

Importantly, most associations originated from individual cohorts, with limited adjustment for nutritional status, renal function, medication exposure, physiological stress, systemic inflammation, and other potential confounders. Multiple exploratory comparisons and the absence of independent replication further restrict interpretation.

Consequently, although selected circulating neuroactive mediators may reflect aspects of GC burden, none has been independently validated as a reliable marker of tumor stage or aggressiveness.

### 3.6. Treatment-Associated Changes and Prognostic Evidence

Longitudinal investigations evaluated circulating motilin, GAL, CCK, PYY, and obestatin following gastric surgery, whereas ProGRP was assessed during chemotherapy. Formal prognostic evidence was considerably more limited and focused primarily on metanephrine and normetanephrine. Importantly, treatment-associated concentration changes should not be interpreted as evidence of tumor-specific response or prognostic utility without appropriate clinical validation.

#### 3.6.1. Changes Following Surgical Treatment

Circulating motilin decreased markedly immediately after subtotal gastrectomy, then recovered toward preoperative concentrations within the first postoperative week [32]. This transient response reflects the strong influence of gastrointestinal anatomy, motility, nutritional intake, and perioperative physiology on motilin secretion, rather than tumor-specific treatment monitoring.

In contrast, circulating GAL increased following tumor resection, approaching concentrations observed in healthy controls [17]. Together with its inverse associations with tumor size, nodal involvement, and TNM stage, this postoperative change suggests a relationship with clinical disease burden. However, GAL trajectories were not evaluated against residual disease, recurrence, or survival, precluding conclusions regarding postoperative surveillance utility.

Thus, both mediators demonstrated treatment-associated changes, but neither has been validated as a marker of surgical efficacy or subsequent oncological outcomes.

#### 3.6.2. Perioperative Changes in CCK, PYY, and Obestatin

Perioperative studies demonstrated changes in circulating CCK, PYY, and obestatin following gastric resection, although these findings primarily reflect gastrointestinal physiological adaptation rather than validated oncological monitoring.

Early investigations documented altered CCK responses after gastrectomy, including changes in meal-stimulated secretion and gallbladder function [25,26]. These publications represent overlapping or complementary clinical observations rather than independent replication.

Postoperative PYY3-36 responses were influenced by vagal preservation and surgical conditions [28], while additional investigations documented short-term changes in PYY3-36 and obestatin following gastric resection [29]. Serial measurements also demonstrated postoperative obestatin alterations after distal gastrectomy [30].

Because gastrectomy simultaneously removes the tumor and modifies nutrient transit, vagal signaling, and enteroendocrine physiology, these changes cannot be attributed specifically to reduced tumor burden. Consequently, CCK, PYY, and obestatin remain markers of perioperative physiological responses rather than validated indicators of residual disease, recurrence, or surgical treatment efficacy.

#### 3.6.3. Changes Following Systemic Treatment

ProGRP showed a marked treatment-associated decrease following three cycles of fluoropyrimidine- and cisplatin-based chemotherapy in patients with advanced GC [33]. The reported concentrations declined from 249.3 ± 28.9 to 26.1 ± 12.8 pg/mL (*p* < 0.001), suggesting substantial longitudinal responsiveness. However, the pretreatment value should not be assumed to represent a separately calculated baseline for the chemotherapy subgroup without explicit confirmation from the original publication.

Despite this pronounced change, the study did not establish whether baseline ProGRP or its longitudinal decline predicted objective chemotherapy response, disease progression, or survival. Moreover, independent validation is lacking. Therefore, ProGRP should be considered a preliminary treatment-responsive circulating marker rather than a validated predictive biomarker.

#### 3.6.4. Survival Associations of Catecholamine Metabolites

The principal survival analysis of circulating catecholamine metabolites in GC identified divergent findings for metanephrine and normetanephrine [15]. Metanephrine was not significantly associated with two-year survival at the evaluated cutoff of 65 pg/mL (HR = 1.462; log-rank *p* = 0.1487). In contrast, higher normetanephrine concentrations were associated with poorer survival at a cutoff of 196 pg/mL (HR = 2.289; log-rank *p* = 0.0104), with two-year survival of 42.30% versus 66.67% in the higher- and lower-concentration groups, respectively.

Although this finding is consistent with associations between elevated normetanephrine and advanced clinicopathological characteristics, comprehensive multivariable adjustment did not establish its prognostic independence. Consequently, normetanephrine represents an exploratory adverse survival-associated marker rather than an independently validated prognostic biomarker.

#### 3.6.5. Lack of Prognostic Validation for Other Mediators

For most circulating neuroactive mediators, formal associations with overall survival, progression-free survival, disease-free survival, or recurrence remain unavailable or insufficiently characterized. This applies to GAL, DA, NPY, motilin, SP, β-endorphin, somatostatin, CCK, NT, VIP, PYY, and obestatin.

Although GAL increased following tumor resection and ProGRP decreased during chemotherapy, neither finding establishes prognostic utility or the ability to predict recurrence or progression [8,10]. Similarly, perioperative changes in CCK, PYY, and obestatin primarily reflect gastrointestinal physiological adaptation and cannot be interpreted as validated oncological surveillance signals [25,26,28,29,30,34,35].

Recent mechanistic evidence implicating serotonin-dependent GPX4 serotonylation and ferroptosis resistance in GC provides biological plausibility but does not establish prognostic performance for circulating 5-HT [5]. Thus, prospective outcome-based validation remains necessary before these mediators can be considered prognostic biomarkers.

#### 3.6.6. Overall Longitudinal and Prognostic Evidence

Overall, several circulating neuroactive mediators demonstrated treatment-associated changes, but their clinical interpretation remains uncertain. Postoperative alterations in motilin, CCK, PYY, and obestatin are strongly influenced by gastrointestinal physiological adaptation [25,26,28,29,30,32], whereas increased GAL following resection and decreased ProGRP during chemotherapy suggest potential disease- or treatment-associated responsiveness without validated monitoring utility [17,33].

Prognostic evidence was particularly limited. Higher normetanephrine, but not metanephrine, was significantly associated with poorer survival in one cohort; however, independent prognostic value and external reproducibility remain unestablished [15].

These findings underscore the distinction between treatment-associated biomarker changes, objective response prediction, longitudinal monitoring, and prognostic validity. Future studies should incorporate standardized serial sampling, objective treatment-response criteria, recurrence and survival endpoints, multivariable adjustment, and independent validation to determine whether circulating neuroactive mediators provide incremental clinical information beyond established prognostic factors.

The available longitudinal and prognostic findings are summarized in Table 1.

### 3.7. Translational Evidence Mapping and Major Evidence Gaps

To place the clinical findings in a broader biological context, we mapped circulating evidence against available tissue-level, receptor, and experimentally supported signaling data relevant to GC. This translational comparison revealed substantial heterogeneity in the degree of convergence between circulating biomarker observations and tumor-associated mechanistic evidence. For some mediators, alterations in blood coexist with corresponding receptor, signaling, or experimental findings, whereas for others, circulating changes remain biologically difficult to interpret. Conversely, several neuroactive pathways with experimental relevance to GC have not yet been investigated as circulating biomarkers in eligible human studies. The relationships between circulating findings, mechanistic evidence, and major translational gaps are summarized in Supplementary Table S3.

#### 3.7.1. Mediators with Convergent Circulating and Mechanistic Evidence

GAL represents one of the clearest examples of convergence between circulating and tumor-associated evidence. Two independent clinical studies showed lower circulating GAL concentrations in patients with GC [17,18], while tumor-level alterations in the GAL/GalR system, associations with clinicopathological severity, and postoperative increases were documented in one cohort [17]. Together, these findings suggest a potentially relevant relationship between systemic GAL alterations and tumor-associated neuropeptide signaling. Nevertheless, the causal relationship between reduced circulating GAL and tumor biology remains unresolved, and the clinical findings require validation in larger independent cohorts.

Serotonin represents another, although considerably more complex, example. Historical blood-based studies investigated 5-HT alterations in GC [10,11,12], whereas contemporary plasma metabolomic evidence demonstrated increased circulating 5-HT [5]. These findings should not be interpreted as defining a single directional GC phenotype because whole blood and plasma represent analytically and biologically distinct compartments, with platelet-related influences and pre-analytical variability further complicating comparisons.

Importantly, recent experimental evidence provides a mechanistic connection between 5-HT and GC progression. Bai et al. demonstrated that serotonin promoted TGM2-mediated serotonylation, stabilized GPX4, increased resistance to ferroptosis, and facilitated gastric tumorigenesis [5]. Although these findings support a biologically coherent tumor-promoting pathway, they do not establish that circulating 5-HT reliably reflects intratumoral serotonergic activity or possesses validated diagnostic or prognostic utility.

DA also shows evidence across circulating and tumor-associated compartments. Amjadi et al. reported substantially reduced plasma dopamine together with alterations in dopamine-related molecular machinery in peripheral and tumor-associated compartments [14]. Although the circulating finding has not been independently replicated, these cross-compartment observations support further investigation of the dopaminergic axis without establishing a direct tumor origin for circulating DA alterations. Whether circulating dopamine reflects tumor biology, systemic neuroendocrine responses, altered metabolism, or a combination of these processes remains uncertain.

NT provides another relevant example of cross-level evidence. Increased circulating NT was positively associated with MMP-9 activity, while experimental analyses implicated NTSR1-dependent PKC, ERK, and PI3K signaling in MMP-9 activation, migration, and invasion [22]. Subsequent experimental work further supported NTSR1 involvement in invasive GC biology [23]. Thus, the NT/NTSR1 axis has both circulating and mechanistic support, although its clinical relevance remains preliminary because independent validation of circulating NT and its associations with disease severity or patient outcomes is lacking.

The GRP/ProGRP pathway similarly combines clinically informative circulating observations with a biologically plausible receptor system. Serum ProGRP was markedly elevated in GC, associated with multiple indicators of tumor burden, and decreased substantially during chemotherapy [33]. These clinical observations can be interpreted in the context of GRP/bombesin receptor biology. However, the measured circulating analyte was ProGRP rather than mature GRP. This distinction precludes directly extrapolating experimental evidence of GRP receptor signaling to the biological activity or tumor origin of circulating ProGRP.

Overall, these pathways illustrate varying degrees of convergence between circulating biomarker observations and tumor-associated mechanisms. Nevertheless, mechanistic plausibility should not be equated with validated diagnostic, treatment-monitoring, or prognostic performance.

#### 3.7.2. Clinical Signals with Limited Mechanistic Integration

Several mediators demonstrated circulating alterations without an equally developed mechanistic framework linking blood concentrations to GC-specific biology. Motilin is a prominent example. Independent studies reported increased circulating motilin [24,32], providing more clinical replication than many other peptides. However, fasting status, gastrointestinal motility, vagal regulation, nutrient exposure, and perioperative physiological changes strongly influence motilin. Therefore, these circulating alterations cannot currently be attributed specifically to tumor-derived mechanisms.

Similarly, increased somatostatin-like immunoreactivity [31], increased SP and β-endorphin [19], reduced NPY [20], reduced CCK [21,27], and increased obestatin [18] provide evidence of systemic neuroactive or neuroendocrine alterations in patients with GC. However, their relationships with tumor-associated receptor signaling and GC-specific biological processes remain insufficiently characterized.

CCK, PYY, and obestatin particularly illustrate the challenges of distinguishing tumor-associated biomarker alterations from gastrointestinal physiological responses. Reduced circulating CCK has been reported in case–control investigations [21,27], while earlier perioperative studies demonstrated that CCK release is substantially altered by gastrectomy [25,26]. Similarly, circulating PYY and obestatin have been investigated in case–control and postoperative settings, with findings indicating sensitivity to gastric resection, vagal regulation, and gastrointestinal adaptation [18,21,28,29,30]. Consequently, alterations in these mediators may reflect changes in gastrointestinal anatomy, nutrient transit, enteroendocrine function, and metabolic regulation rather than tumor burden alone.

For NPY, the association between lower circulating concentrations, more advanced disease, larger tumors, and weight loss raises the possibility that the biomarker signal partly reflects cancer-associated nutritional and metabolic alterations rather than direct tumor production [20]. This interpretation further emphasizes the need to account for nutritional status and systemic metabolic disturbances when evaluating circulating neuroactive mediators in GC.

Metanephrine and normetanephrine occupy an intermediate position. Their associations with tumor size, T and N categories, TNM stage, and, for normetanephrine, survival suggest a relationship with disease severity [15]. However, these analytes are catecholamine metabolites rather than direct receptor-active neurotransmitters, and circulating concentrations may reflect systemic sympathoadrenal activation rather than tumor-derived catecholaminergic signaling. Furthermore, the reported survival association for normetanephrine was based on univariable analysis, and its independent prognostic value remains unestablished.

Collectively, these findings indicate that circulating neuroactive alterations in GC may integrate tumor-associated, neural, gastrointestinal, endocrine, and metabolic processes. Distinguishing these contributions will require standardized pre-analytical protocols, appropriately matched control populations, mechanistically informed clinical investigations, and independent validation.

#### 3.7.3. Mechanistically Relevant Pathways Lacking Circulating Human Evidence

The translational mapping also identified several neuroactive pathways with experimental or tissue-level relevance for which the systematic search did not identify corresponding eligible circulating human GC studies.

GABAergic signaling represents one such example. Experimental studies have investigated GABA receptors and related signaling in gastric cancer cells or tissues, but no eligible study in the present systematic review quantified circulating GABA in patients with GC. Accordingly, the biological relevance of GABAergic signaling remains distinct from its unestablished clinical applicability as a circulating GC biomarker.

A comparable evidence gap was identified for PACAP and CGRP. Although PACAP-related receptor systems, including PAC1 and the shared VPAC receptors, provide a plausible connection between neuropeptide signaling and gastrointestinal tumor biology, no eligible circulating human GC studies were identified for PACAP or CGRP.

No eligible circulating human GC studies were identified for NMU or CART peptide. Importantly, the absence of eligible circulating studies should be interpreted as a lack of clinical biomarker evidence, not as evidence of biological inactivity or an established tumor-promoting role in GC.

These findings reveal an important mismatch between mechanistic research priorities and circulating biomarker development. Pathways investigated experimentally have not necessarily undergone clinical validation, whereas some repeatedly reported circulating alterations, such as those involving motilin, lack comparably developed tumor-specific mechanistic explanations.

#### 3.7.4. Pre-Analytical and Analytical Heterogeneity as a Translational Barrier

Evidence mapping identified heterogeneity in pre-analytical and analytical procedures as a major cross-cutting limitation. This issue is particularly important for serotonin. Because a substantial proportion of peripheral 5-HT is associated with platelets, whole blood, serum, and platelet-poor plasma represent biologically and analytically distinct matrices. Platelet release during clotting may substantially influence serum measurements, whereas plasma concentrations depend on the extent of platelet removal and sample-processing procedures.

Consequently, the historical whole-blood findings [10,11,12] and contemporary plasma findings [5] should not be considered directly interchangeable. Differences in biological matrix, platelet handling, collection and processing procedures, analytical platforms, and study populations may contribute to apparently discordant circulating 5-HT observations.

Similar concerns apply to other neuroactive mediators. Catecholamines and their metabolites are sensitive to physiological stress, posture, fasting, medication exposure, and sampling conditions. Gut–brain peptides such as motilin, CCK, PYY, and obestatin are influenced by fasting–feeding status, gastrointestinal physiology, vagal signaling, and previous gastric surgery. For neuropeptides, proteolytic degradation and differences in sample collection, processing delays, centrifugation, storage conditions, and freeze–thaw exposure may introduce additional analytical variability.

Reporting of these variables was inconsistent across the included literature, particularly among older studies. The transition from historical radioimmunoassays to contemporary immunoassays and mass-spectrometry-based techniques further complicates comparisons of absolute biomarker concentrations across studies. Standardization of both pre-analytical and analytical procedures is therefore a prerequisite for reproducible validation of circulating neuroactive biomarkers in GC.

To further characterize these limitations, a mediator-specific assessment of physiological and pre-analytical confounding was conducted using the methodological information available from the included publications (Supplementary Table S6). Among the three assessable serotonin studies, the biological matrix was identifiable in each publication, comprising whole blood, serum, and plasma measurements. However, explicit information regarding platelet counts, platelet activation, and residual platelet contamination could not be consistently verified. Bai et al. [5] documented a two-step plasma centrifugation procedure and storage at −80 °C, although these procedures alone do not establish quantitative control of platelet-related interference.

For gastrointestinal peptides, reporting completeness varied according to mediator and study design. Among the three motilin publications, Zhang et al. [32] documented perioperative sampling intervals, including postoperative days 1 and 7. For CCK, four publications were identified, including investigations documenting fasting conditions, standardized meal stimulation, and physiological responses following gastrectomy [21,25,26,27]. However, the potentially overlapping reports by Inoue et al. [25] and Huang et al. [26] should not be interpreted as independent clinical evidence without further verification. Among three PYY publications, fasting conditions were documented in selected investigations, while postoperative changes were evaluated in relation to gastric surgery and vagal preservation [21,28,29]. Similarly, two of the three obestatin publications investigated postoperative alterations [29,30]. These observations indicate that surgery-related physiological changes constitute an important alternative explanation for circulating peptide variations.

Additional examples of pre-analytical documentation were identified for other mediator classes. Mehedințeanu et al. [15] described fasting blood collection, avoidance of alcohol and caffeine before sampling, and precautions regarding potentially interfering medications when assessing catecholamine metabolites. Zhang et al. [17] reported fasting blood collection, the use of prechilled EDTA tubes containing aprotinin, and controlled plasma storage for galanin measurements. These methodological details demonstrate that relevant confounding variables were addressed in selected studies, although comparable information was not consistently accessible across the broader literature.

Importantly, the reporting frequencies summarized in Supplementary Table S6 represent minimum confirmed frequencies based on accessible source information rather than exhaustive estimates of methodological compliance. Variables that could not be verified should not automatically be regarded as uncontrolled. Nevertheless, incomplete methodological documentation limits cross-study comparability and reduces confidence that circulating biomarker differences are independent of physiological, surgical, or analytical influences. These findings reinforce the need for standardized mediator-specific sampling protocols and prospective validation before clinical implementation.

#### 3.7.5. Major Evidence Gaps

The evidence map identified several recurrent limitations across the literature. First, independent clinical replication remains insufficient for most circulating neuroactive mediators. GAL and motilin represent notable examples of replicated directional circulating findings [17,18,24,32], while CCK has also been investigated in more than one case–control context [21,27]. Nevertheless, the generally modest sample sizes heterogeneous analytical methods, and differences in clinical populations limit the strength of the available evidence.

Second, many investigations used healthy individuals as the principal comparator rather than patients with benign or premalignant gastric disease. This design limits assessment of clinical specificity and may overestimate diagnostic discrimination. ProGRP was notable because it was evaluated against both healthy controls and patients with benign gastric disease [33]. Future diagnostic investigations should incorporate clinically relevant comparator populations to determine whether circulating neuroactive mediators can distinguish GC from nonmalignant gastric conditions.

Third, evidence specifically addressing early-stage disease remains limited. Some studies focused on advanced GC, whereas others lacked sufficiently granular stage-specific analyses. Although several perioperative investigations enrolled patients with early GC [25,26,28,29,30], their primary objectives concerned physiological responses to gastrectomy rather than early cancer detection. Consequently, the diagnostic performance of circulating neuroactive mediators in early-stage GC remains insufficiently established.

Fourth, several clinical settings have documented longitudinal biomarker alterations, but their oncological significance remains uncertain. Surgical or perioperative changes have been described for motilin, GAL, CCK, PYY, and obestatin [17,25,26,28,29,30,32], and ProGRP was evaluated during systemic chemotherapy [33]. However, postoperative alterations in gut–brain peptides may predominantly reflect gastrointestinal anatomical and physiological adaptation rather than tumor burden. Future studies should relate serial biomarker measurements to objective treatment response, recurrence, disease progression, and survival.

Fifth, formal prognostic evidence is particularly sparse. Among the included circulating studies, the clearest survival analysis concerned metanephrine and normetanephrine, with a statistically significant adverse survival association for higher normetanephrine but not metanephrine [15]. However, this association was identified in univariable analysis and has not been established as an independent or externally validated prognostic effect. Thus, clinicopathological association, postoperative normalization, or treatment-associated decline should not be used as a surrogate for demonstrated prognostic value.

Sixth, multivariable validation was limited. Associations between neuroactive mediators and stage, treatment response, or survival may be confounded by age, nutritional status, renal function, medication exposure, psychological and physiological stress, systemic inflammation, pain, cachexia, treatment, and other disease-related factors. The limited adjustment for these variables, together with exploratory multiple comparisons, reduces confidence in the specificity and reproducibility of reported associations.

Finally, integration between circulating biomarker measurement and matched tumor biology remained limited. Few investigations simultaneously evaluated circulating concentrations, tumor-associated receptor expression, downstream signaling, and longitudinal clinical outcomes within the same patient population. Such paired and longitudinal designs would be particularly valuable for determining whether circulating neuroactive alterations originate from, respond to, or merely coexist with tumor-associated neural and neuroendocrine signaling.

Overall, the translational evidence map demonstrates a substantial disconnect between mechanistic neuro–tumor biology and clinical circulating biomarker development. GAL, 5-HT, DA, NT/NTSR1, and the GRP/ProGRP axis currently provide examples in which circulating observations can be considered alongside tumor-level or experimental evidence, although the strength and nature of this convergence differ considerably among mediators. In contrast, GABA, PACAP, CGRP, NMU, and CART represent pathways of potential mechanistic interest for which eligible circulating human GC evidence remains unavailable.

Figure 2 illustrates the proposed relationships among biological sources, circulating neuroactive mediators, receptor-mediated signaling, downstream tumor-associated effects, and potential clinical applications. The mediator-specific translational evidence and principal evidence gaps are summarized in Supplementary Table S3.

#### 3.7.6. Risk-of-Bias and Methodological Quality Assessment

The study-level methodological appraisal revealed considerable heterogeneity in study design, participant selection, biomarker assessment, and reporting completeness (Supplementary Table S5). The interpretability of risk-of-bias judgments varied according to the availability of methodological information, particularly between studies with accessible full texts and historical publications for which only abstracts or bibliographic records were available.

Across the observational studies, the principal methodological concerns involved the representativeness and comparability of study populations, the identification and control of potential confounding factors, and the completeness of laboratory and statistical reporting. Case–control comparisons were particularly susceptible to selection-related limitations, while studies involving perioperative measurements required additional consideration of surgical stress, sampling intervals, and changes in physiological status. For investigations reporting diagnostic performance, the use of selected cancer and healthy-control populations, together with incompletely documented diagnostic thresholds or independent validation procedures, limited the interpretation of reported sensitivity, specificity, and discriminatory performance. These concerns should not be interpreted as uniformly demonstrated high risk of bias across all studies, because several domains remained insufficiently reported.

The complementary analytical and pre-analytical appraisal identified inconsistencies in the reporting of biological matrices, sampling conditions, specimen processing, storage procedures, and assay characteristics. Although assay platforms were identifiable in several publications, information concerning analytical precision, calibration, quality-control procedures, and other determinants of measurement reproducibility was not consistently available. Such reporting limitations were particularly relevant when comparing circulating concentrations of matrix-sensitive mediators, including serotonin, or interpreting differences across studies using distinct analytical methods.

For studies available only through abstracts, risk-of-bias judgments were necessarily restricted to the methodological information explicitly reported, and insufficiently documented domains were classified as unclear. Historical publications available exclusively as bibliographic records, without adequate information to verify eligibility or assess methodological quality, were considered not assessable and did not contribute to the qualitative evidence synthesis. Accordingly, the available evidence supports a cautious interpretation of the reported associations between circulating neuroactive mediators and gastric cancer, particularly regarding their diagnostic, prognostic, and clinical applicability. Detailed study-level assessments and explanatory notes are provided in Supplementary Table S5.

## 4. Discussion

### 4.1. Integrated Biological Interpretation of Circulating Neuroactive Alterations in Gastric Cancer

This systematic review integrates more than five decades of clinical research on circulating neurotransmitters, neuropeptides, and related neuroactive mediators in gastric cancer (GC). The central finding is that circulating neuroactive alterations are detectable in GC, but their interpretation requires distinguishing tumor-associated signaling from systemic neuroendocrine responses and gastrointestinal physiological adaptations. The available evidence remains heterogeneous, and no investigated mediator has yet achieved sufficient validation for routine clinical application.

At the tumor–microenvironment level, neuroactive mediators may participate in receptor-dependent autocrine or paracrine signaling involving malignant cells, neural structures, stromal compartments, and immune populations [2,3]. Experimental evidence supports several potentially relevant pathways. Serotonin has been implicated in TGM2-mediated serotonylation, GPX4 stabilization, and ferroptosis resistance [5], while NT/NTSR1 signaling has been associated with migration and invasion through downstream pathways involving PKC, ERK, PI3K, and MMP-9 [22,23]. Similarly, SP/NK-1R signaling has demonstrated proliferative and migratory effects in experimental GC models [34,35]. These mechanisms provide biological context for selected circulating observations but do not establish that blood concentrations directly reflect intratumoral pathway activity.

GAL and ProGRP illustrate the distinction between reproducible clinical associations and mechanistic interpretation. Two independent GC cohorts reported reduced circulating GAL [17,18], with one investigation also noting clinicopathological and postoperative associations [17]. ProGRP demonstrated diagnostic discrimination, relationships with tumor burden, and chemotherapy-associated changes within a single clinical study [33]. Nevertheless, the evidence supporting these candidates differs substantially in replication and clinical validation. Moreover, circulating ProGRP should not be equated with mature GRP or used as direct evidence of GRP/GRPR-mediated tumor signaling.

A second biological dimension involves systemic neural and neuroendocrine adaptation. Reduced circulating dopamine and alterations in dopamine-related molecular components [14], together with associations involving metanephrine and normetanephrine [15], suggest disturbances in catecholaminergic regulation. These findings may reflect interactions among autonomic activity, physiological stress, metabolic changes, and malignancy-related processes. Although higher normetanephrine was associated with poorer survival in univariable analysis, its independent prognostic significance remains unconfirmed [15]. Consequently, circulating catecholaminergic alterations cannot be interpreted as direct indicators of tumor-local neurotransmitter activity.

A third dimension concerns gastrointestinal neuroendocrine physiology. Replicated alterations involving motilin and CCK [21,24,27,32], together with postoperative changes in CCK, PYY, obestatin, and other gut–brain peptides [25,26,28,29,30,32], indicate substantial physiological responsiveness. However, gastric resection, altered nutrient transit, vagal signaling, and enteroendocrine adaptation may influence these mediators independently of residual tumor burden. Treatment-associated changes therefore cannot be assumed to demonstrate oncological monitoring utility.

Serotonin additionally highlights the importance of biological matrix and analytical context. Historical whole-blood observations and contemporary plasma findings differ in direction [5,10,11,12], but these measurements represent distinct peripheral compartments influenced by platelet content and sample handling. Accordingly, apparent discrepancies should not be interpreted as direct biological contradictions without considering pre-analytical and methodological differences.

Overall, the circulating neuroactive profile in GC is best understood as a potential combination of tumor-associated signaling, systemic neuroendocrine adaptation, and gastrointestinal physiological responses. These mechanisms are not mutually exclusive, and their relative contributions remain insufficiently characterized. The principal translational challenge is therefore to establish which circulating alterations reflect tumor-specific biology, which represent broader host responses, and whether either category provides clinically meaningful information beyond established biomarkers.

### 4.2. Clinical Relevance and Biomarker Validation

The clinical evidence identifies several candidates at different stages of biomarker development rather than a uniformly validated neuroactive signature. GAL and motilin have demonstrated replicated directional alterations [17,18,24,32], while CCK has shown concordant reductions in independent clinical investigations [21,27]. ProGRP offers comparatively detailed diagnostic and clinicopathological characterization within a single study [33]. Other mediators, including 5-HT, DA, NT, and SP, are particularly relevant for investigating relationships between circulating observations and tumor-associated molecular pathways [5,14,19,22,23,25,34,35].

Nevertheless, case–control differences do not establish clinical diagnostic utility. Most studies included limited sample sizes, and comparisons with healthy controls predominated over clinically relevant benign or premalignant gastric conditions. ProGRP was evaluated against both healthy participants and patients with benign gastric disease [33], but its diagnostic performance requires independent confirmation. Early-stage discrimination, reproducible thresholds, and incremental performance beyond conventional tumor markers remain insufficiently established.

The distinction between diagnostic, monitoring, predictive, and prognostic applications is equally important. Postoperative changes in GAL and gut–brain peptides, or chemotherapy-associated decreases in ProGRP, demonstrate biological or treatment-associated responsiveness without necessarily establishing tumor-specific monitoring performance [17,25,26,28,29,30,32]. Similarly, the reported survival association for normetanephrine remains preliminary because comprehensive multivariable and external validation are lacking [15].

Future clinical assessment should therefore prioritize reproducibility, clinically appropriate comparator groups, stage-specific diagnostic performance, longitudinal associations with objective outcomes, and independent validation. Until these requirements are met, circulating neuroactive mediators should be regarded as exploratory candidates rather than established diagnostic or prognostic biomarkers in GC.

### 4.3. Pre-Analytical and Analytical Standardization as a Priority

The development of circulating neuroactive biomarkers in GC requires rigorous control of biological matrix, sample handling, and analytical methodology. Serum, plasma, and whole blood may represent biologically distinct analyte pools rather than interchangeable sample types. This distinction is particularly important for serotonin, whose peripheral concentrations are strongly influenced by platelet content and activation. Consequently, historical whole-blood findings [10,11,12] cannot be directly compared with contemporary plasma metabolomic measurements [5].

Pre-analytical protocols should standardize fasting status, sampling time, posture, medication exposure, physiological stress, anticoagulant use, processing delays, centrifugation, storage, and freeze–thaw conditions. Platelet depletion is especially relevant for 5-HT, whereas catecholamine-related analytes require careful control of stress-related factors. Gut–brain peptides, including motilin, CCK, PYY, and obestatin, additionally require standardized nutritional and gastrointestinal conditions.

Analytical heterogeneity further limits comparability across studies spanning more than five decades. Historical radioimmunoassays and contemporary immunoassays or mass-spectrometry-based platforms differ in specificity, calibration, sensitivity, and molecular species detected. Future investigations should report assay characteristics, detection limits, precision, calibration procedures, and quality-control measures. Mass-spectrometry-based analyses should additionally document internal standards, extraction recovery, and matrix effects.

Harmonized pre-analytical and analytical procedures are essential for multicenter replication, reproducible diagnostic thresholds, and meaningful clinical validation.

### 4.4. A Translational Roadmap and Priorities for Future Clinical Studies

Future research should progress from exploratory case–control observations toward prospective, multicenter, pathway-oriented biomarker validation. Study designs should incorporate standardized blood sampling, clinically relevant comparator populations, and predefined diagnostic or prognostic objectives. Controls should include benign inflammatory or ulcerative gastric conditions and premalignant lesions, while GC cohorts should represent different TNM stages, with deliberate inclusion of early-stage disease.

Candidate selection should reflect the maturity and biological relevance of existing evidence. GAL, motilin, and CCK warrant independent confirmation of their replicated circulating alterations [17,18,21,24,27,32]. ProGRP requires external validation of its diagnostic and clinicopathological associations [33]. Mechanistically relevant pathways involving 5-HT, DA, SP/NK-1R, and NT/NTSR1 offer opportunities for integrated circulating and tumor-level investigations [5,14,19,22,23,34,35]. Catecholamine metabolites, particularly normetanephrine, require prospective assessment of their potential prognostic relevance [15]. Mediators lacking eligible circulating human GC evidence, including GABA, PACAP, CGRP, NMU, and CART, should remain exploratory candidates rather than presumed biomarkers.

Three complementary priorities should guide clinical translation. First, diagnostic studies should establish stage-specific discrimination using clinically appropriate controls and report validated thresholds, AUCs with confidence intervals, sensitivity, and specificity. Second, longitudinal investigations should relate biomarker trajectories to objective treatment response, recurrence, progression-free survival, and overall survival, while distinguishing tumor-related changes from postoperative physiological adaptation. Third, paired blood–tumor studies should examine whether circulating concentrations correspond to receptor expression, downstream signaling, and tumor-associated neural or immune interactions.

Multivariable biomarker models should evaluate incremental clinical value beyond TNM stage, histopathological characteristics, conventional tumor markers, and relevant systemic confounders. Candidate multimarker signatures should undergo internal and independent external validation, with assessment of discrimination, calibration, and clinical utility.

Ultimately, the objective should not be to establish every neuroactive mediator as an independent GC biomarker, but to identify reproducible circulating signatures that capture clinically relevant aspects of tumor-associated signaling and host neuroendocrine responses. Figure 2 illustrates the proposed biological and translational relationships, while Supplementary Table S3 summarizes mediator-specific evidence and research gaps.

### 4.5. Strengths and Limitations

This systematic review provides a broad synthesis of circulating neuroactive mediators investigated in GC and distinguishes clinical biomarker observations from complementary tissue-level and experimental evidence. Its principal strengths include the integration of multiple neurotransmitter and neuropeptide families, the differentiation of diagnostic, clinicopathological, treatment-associated, and prognostic findings, and the identification of translational evidence gaps.

Several limitations should be acknowledged. First, the primary evidence base comprised relatively few studies for most mediators, frequently involving small cohorts and limited independent replication. Second, differences in disease stage, comparator populations, study design, and outcome reporting restricted direct comparisons and precluded meaningful quantitative pooling for most biomarkers. Third, historical variation in biological matrices, analytical platforms, and incompletely reported pre-analytical procedures introduced substantial methodological heterogeneity.

Fourth, systemic confounding was insufficiently controlled in many investigations. Stress, pain, nutritional status, gastrointestinal physiology, renal and hepatic function, inflammation, medication exposure, and cancer treatment may independently influence circulating neuroactive concentrations. Fifth, publication bias, selective outcome reporting, and exploratory multiple comparisons may have contributed to overestimation of individual associations. Sixth, formal prognostic analyses, multivariable adjustment, and external validation were generally limited.

An additional limitation concerns the methodological quality and completeness of reporting across the included studies. As detailed in Supplementary Table S5, substantial variability was observed in participant selection, control of potential confounding factors, analytical procedures, and pre-analytical reporting. Furthermore, several historical and non-English-language publications were accessible only through abstracts, restricting the assessment of individual risk-of-bias domains. Two historical publications for which neither an informative abstract nor a full text was available could not be adequately assessed and were therefore retained in the source audit but excluded from the qualitative evidence synthesis. Importantly, insufficient methodological reporting was not equated with demonstrated high risk of bias. These limitations reduce confidence in the comparability and reproducibility of the reported findings and underscore the need for prospective, adequately powered studies incorporating standardized sampling protocols, validated analytical methods, appropriate control groups, and transparent reporting of methodological quality.

An important limitation of the available evidence is the mediator-specific susceptibility to physiological and pre-analytical confounding. For serotonin, platelet-related effects and differences between serum, plasma, and whole-blood measurements complicate comparisons across studies and may contribute to apparently divergent findings. For motilin and CCK, fasting conditions, gastrointestinal function, and surgery-related physiological changes represent particularly relevant sources of variability, especially in longitudinal and perioperative investigations. Inadequate reporting of these variables limits the ability to distinguish tumor-associated biomarker alterations from systemic or gastrointestinal physiological responses. Consequently, observed changes in circulating concentrations should not automatically be interpreted as direct indicators of tumor activity, treatment response, or clinical prognosis. Standardized mediator-specific sampling protocols, detailed reporting of relevant confounders, and appropriately controlled prospective investigations are required to establish the robustness and clinical applicability of these findings.

An additional methodological limitation concerns the retrospective registration of this systematic review. Although the review methodology was subsequently documented in the Open Science Framework, registration occurred after the completion of study selection, data extraction, and evidence synthesis. Therefore, the registration record does not provide prospective verification of the originally intended eligibility criteria, outcomes, or analytical decisions, and potential deviations from an initial review plan cannot be independently assessed. This limitation should be considered alongside the incomplete accessibility of several historical publications and uncertainties regarding selected study-level characteristics, including sample size, cohort independence, and treatment-subgroup comparability. These constraints were explicitly documented in the evidence tables and supplementary methodological assessments rather than resolved through unsupported assumptions. Accordingly, the findings should be interpreted as a transparent synthesis of the available evidence, without implying that all methodological decisions were prospectively specified or that all study-level uncertainties could be resolved.

Finally, Embase could not be searched because institutional access was unavailable, creating a possibility that relevant studies were missed. The supplementary mechanistic evidence was used for biological contextualization rather than treated as additional eligible circulating clinical evidence.

These limitations restrict causal interpretation and clinical generalizability. The available findings should therefore be considered hypothesis-generating and supportive of further prospective validation, rather than sufficient for routine clinical implementation.

## 5. Conclusions

This systematic review highlights the emerging relevance of circulating neuroactive mediators in gastric cancer while revealing substantial gaps between biological plausibility, clinical associations, and validated biomarker applications. The translational evidence map indicates that circulating alterations may reflect overlapping processes involving tumor-associated receptor signaling, systemic neuroendocrine and stress responses, and gastrointestinal physiological adaptations, rather than exclusively tumor-specific mechanisms. Although complementary clinical and experimental evidence supports selected neuroactive pathways, the correspondence between peripheral mediator concentrations and intratumoral signaling remains insufficiently established. Limited independent replication, heterogeneous analytical methodologies, systemic confounding, and insufficient prospective validation currently preclude routine clinical implementation. Future investigations should prioritize standardized, multicenter studies integrating circulating measurements with matched tumor biology, clinically relevant comparator populations, and longitudinal outcomes. Ultimately, pathway-oriented multimarker approaches may provide a more biologically coherent framework for identifying clinically informative neuroactive signatures and advancing the translation of neuro–tumor interactions into gastric cancer biomarker research.

## Figures and Tables

**Table 1:** Treatment-associated changes and prognostic evidence for circulating neuroactive mediators in gastric cancer.

Study	Marker	Clinical Setting	Patients	Longitudinal/Outcome Assessment	Main Finding	Statistical Result	Evidence Category	Interpretation
Inoue et al., 1987 [25] *	CCK	Gastrectomy; early GC	5	CCK release before and after gastrectomy	Gastrectomy-associated alteration in circulating CCK response	Reported	Perioperative physiological response	Demonstrates sensitivity of CCK physiology to gastrectomy; not evidence of tumor-specific monitoring
Huang et al., 1989 [26] *	CCK	Gastrectomy; early GC	5	CCK release before and after gastrectomy	Perioperative alteration in CCK release	Reported	Perioperative physiological response	Overlapping/complementary report to Inoue et al. [28]; not independent replication
Zhang et al., 2008 [32]	Motilin	Subtotal gastrectomy	39	Preoperative, postoperative day 1, and postoperative day 7	Elevated preoperatively; decreased on day 1 and returned toward preoperative concentrations by day 7	Day-1 decrease, *p* < 0.001	Surgical longitudinal response	Rapid dynamics indicate strong influence of perioperative GI physiology
Kim et al., 2009 [30]	Obestatin	Distal gastrectomy; early GC	45	Serial measurements following gastric resection	Circulating obestatin changed following gastrectomy	Reported	Perioperative physiological response	Supports sensitivity to gastric resection and postoperative metabolic/GI adaptation; tumor specificity not established
Kim et al., 2012 [28]	PYY3-36	Subtotal gastrectomy	16	Postoperative PYY assessment according to vagus nerve preservation/vagotomy	Circulating PYY response varied with surgical/vagal conditions	Reported	Perioperative physiological response	Demonstrates strong influence of vagal and postoperative GI physiology
Jeon et al., 2015 [29]	PYY3-36/obestatin	Gastric resection; early GC	NR †	Short-term pre-/post-resection assessment	Postoperative changes in circulating PYY3-36 and obestatin	Reported	Perioperative physiological response	Dynamic response to gastric resection; cannot be attributed specifically to tumor removal
Zhang et al., 2019 [17]	GAL	Surgical resection	34	Pre- vs. postoperative plasma GAL	20.73 ± 2.52 → 32.74 ± 4.15 pg/mL; postoperative value approached healthy-control level (36.27 ± 5.94 pg/mL)	*p* < 0.01	Surgical longitudinal response	Potential tumor-burden-responsive signal; recurrence and survival value not established
Li et al., 2020 [33]	ProGRP	Fluoropyrimidine + cisplatin chemotherapy; advanced GC	108	Before vs. after three treatment cycles	Reported reduction in serum ProGRP concentrations following three chemotherapy cycles: 249.3 ± 28.9 vs. 26.1 ± 12.8 pg/mL ‡	*p* < 0.001	Systemic-treatment monitoring	Treatment-associated concentration difference; paired subgroup comparability and clinical response-monitoring validity not established.
Mehedințeanu et al., 2021 [15]	Metanephrine	Survival analysis	91	Cutoff 65 pg/mL; 2-year survival	Low: 58.33%; high: 48.07%	HR = 1.462; log-rank *p* = 0.1487	Prognostic	No statistically significant survival association at the evaluated cutoff
Mehedințeanu et al., 2021 [15]	Normetanephrine	Survival analysis	91	Cutoff 196 pg/mL; 2-year survival	Low: 66.67%; high: 42.30%	HR = 2.289; log-rank *p* = 0.0104	Prognostic	Significant univariable adverse survival association; independence not established

Notes: Treatment-associated changes in circulating mediator concentrations should not be interpreted as evidence of predictive or prognostic utility unless they are directly associated with objective treatment response or subsequent clinical outcomes. Post-gastrectomy changes in gastrointestinal neuroactive peptides may reflect alterations in gastrointestinal anatomy, nutrient transit, vagal signaling, motility, and enteroendocrine physiology, independently of changes in tumor burden. * Inoue et al. [25] and Huang et al. [26] were treated as potentially overlapping or complementary reports because of similarities in investigators, clinical setting, sample size, and patient characteristics. Cohort independence could not be conclusively verified; therefore, these publications were not interpreted as independent clinical replications. † The sample size for Jeon et al. [29] could not be verified from the accessible source information and is reported as NR. The study contributes descriptive evidence concerning short-term postoperative changes in circulating PYY3-36 and obestatin, but its sample size is not included in quantitative patient-count summaries. ‡ Li et al. [33] reported a reduction in serum ProGRP concentrations from 249.3 ± 28.9 to 26.1 ± 12.8 pg/mL following chemotherapy. Although 108 patients were described in the treatment subgroup, the available reporting does not establish that the pretreatment mean represents a separately calculated baseline value for those same 108 patients. Accordingly, the reported comparison is presented descriptively and should not be interpreted as a verified paired treatment-response estimate.

## Data Availability

The data used to support the findings of this study are available from the corresponding author upon reasonable request.

## Supplementary Materials

The following supporting information can be downloaded at: https://www.mdpi.com/article/10.3390/cancers18213562/s1, Table S1: PRISMA 2020 checklist; Table S2: Complete electronic search strategies and database-specific retrieval results; Table S3: Translational evidence mapping of circulating neuroactive mediators in gastric cancer; Table S4: Integrated evidence on circulating neuroactive mediators in gastric cancer; Table S5: Study-level risk-of-bias and methodological quality assessment of publications investigating circulating neuroactive mediators in gastric cancer; Table S6: Mediator-Specific Physiological and Pre-analytical Confounders, Reporting Completeness, and Implications for Clinical Interpretation in Gastric Cancer. Mediator-Specific Physiological and Pre-analytical Confounders: Source-Confirmed Reporting and Clinical Interpretation. Evidence base: 22 publication records from Supplementary Table S4–S5 with potentially assessable circulating-mediator evidence. The two bibliographic-only historical records (Podil’chak et al., 1970 [11]; Agaev et al., 1973 [12]) are excluded. This table documents confirmed reporting in accessible source material; it does not establish absence of control when information could not be verified.
