# Supplementary material for: Circulating Neurotransmitters, Neuropeptides, and Neuroactive Mediators in Gastric Cancer: A Systematic Review of Diagnostic, Prognostic, and Clinicopathological Evidence
Source: Cancers (Basel). 2026 Nov 6;18(21):3562. doi: 10.3390/cancers18213562 (PMC13761736; doi:10.3390/cancers18213562)
Supplement: Supplementary file 1 [file cancers-18-03562-s001.zip › cancers-4644883-supplementary.pdf]

cancers

MDPI

Table S1. PRISMA 2020 checklist.

Section and Topic	Item #	Checklist item	Location where item is reported
		**TITLE**	
Title	1	Identify the report as a systematic review.	Title.
		**ABSTRACT**	
Abstract	2	See the PRISMA 2020 for Abstracts checklist.	Abstract.
		**INTRODUCTION**	
Rationale	3	Describe the rationale for the review in the context of existing knowledge.	Section 1 (Introduction).
Objectives	4	Provide an explicit statement of the objective(s) or question(s) the review addresses.	Section 1 (Introduction), final paragraph; Section 2.1.
		**METHODS**	
Eligibility criteria	5	Specify the inclusion and exclusion criteria for the review and how studies were grouped for the syntheses.	Sections 2.3 and 2.6.
Information sources	6	Specify all databases, registers, websites, organisations, reference lists and other sources searched or consulted to identify studies. Specify the date when each source was last searched or consulted.	Section 2.2; last-search date to be reconciled with the final manuscript and Supplementary Table S2.
Search strategy	7	Present the full search strategies for all databases, registers and websites, including any filters and limits used.	Section 2.2; full database-specific search strings and retrieval results: Supplementary Table S2 (not S1).
Selection process	8	Specify the methods used to decide whether a study met the inclusion criteria of the review, including how many reviewers screened each record and each report retrieved, whether they worked independently, and if applicable, details of automation tools used in the process.	Section 2.4. Verify and explicitly report reviewer number, independence and screening procedure if documented; do not infer.
Data collection process	9	Specify the methods used to collect data from reports, including how many reviewers collected data from each report, whether they worked independently, any processes for obtaining or confirming data from study investigators, and if applicable, details of automation tools used in the process.	Section 2.5. Verify and explicitly report data extractor number, independence and discrepancy resolution if documented.

Section and Topic	Item #	Checklist item	Location where item is reported
Data items	10a	List and define all outcomes for which data were sought. Specify whether all results that were compatible with each outcome domain in each study were sought (e.g. for all measures, time points, analyses), and if not, the methods used to decide which results to collect.	Section 2.5: diagnostic, clinicopathological, treatment-associated and survival outcome domains.
	10b	List and define all other variables for which data were sought (e.g. participant and intervention characteristics, funding sources). Describe any assumptions made about any missing or unclear information.	Sections 2.5–2.6; unavailable information marked NR or not assessable; assumptions must be explicit.
Study risk of bias assessment	11	Specify the methods used to assess risk of bias in the included studies, including details of the tool(s) used, how many reviewers assessed each study and whether they worked independently, and if applicable, details of automation tools used in the process.	Section 2.7 and Section 3.7.6; study-level appraisal in Supplementary Table S5. Assessor number and independent appraisal procedure require confirmation.
Effect measures	12	Specify for each outcome the effect measure(s) (e.g. risk ratio, mean difference) used in the synthesis or presentation of results.	Sections 2.5 and 2.8; study-level concentrations, diagnostic performance and prognostic estimates where available; no pooled effect estimate.
Synthesis methods	13a	Describe the processes used to decide which studies were eligible for each synthesis (e.g. tabulating the study intervention characteristics and comparing against the planned groups for each synthesis (item #5)).	Sections 2.3, 2.6 and 2.8; mediator- and outcome-based qualitative grouping.
	13b	Describe any methods required to prepare the data for presentation or synthesis, such as handling of missing summary statistics, or data conversions.	Sections 2.5 and 2.8; missing values marked NR; no meta-analytic conversion.
	13c	Describe any methods used to tabulate or visually display results of individual studies and syntheses.	Section 2.8; main evidence tables, Figure 2 and Supplementary Tables S3–S4.
	13d	Describe any methods used to synthesize results and provide a rationale for the choice(s). If meta-analysis was performed, describe the model(s), method(s) to identify the presence and extent of statistical heterogeneity, and software package(s) used.	Section 2.8; structured qualitative synthesis; meta-analysis not undertaken due to heterogeneity.

Section and Topic	Item #	Checklist item	Location where item is reported
	13e	Describe any methods used to explore possible causes of heterogeneity among study results (e.g. subgroup analysis, meta-regression).	Sections 2.8 and 3.7.4; qualitative examination of matrix, assays, comparators, surgical context and confounding; Supplementary Table S6.
	13f	Describe any sensitivity analyses conducted to assess robustness of the synthesized results.	No formal sensitivity analyses reported; this limitation should be stated explicitly.
Reporting bias assessment	14	Describe any methods used to assess risk of bias due to missing results in a synthesis (arising from reporting biases).	No formal synthesis-level assessment of bias due to missing results; discussed in Section 4.5.
Certainty assessment	15	Describe any methods used to assess certainty (or confidence) in the body of evidence for an outcome.	No formal certainty-of-evidence grading (e.g., GRADE) reported.
**RESULTS**			
Study selection	16a	Describe the results of the search and selection process, from the number of records identified in the search to the number of studies included in the review, ideally using a flow diagram.	Section 3.1 and Figure 1 (PRISMA flow diagram). Reconcile publication/eligibility counts with S4–S5 before submission.
	16b	Cite studies that might appear to meet the inclusion criteria, but which were excluded, and explain why they were excluded.	Figure 1 presents exclusion categories; verify whether individually excluded near-eligible reports are cited and justified.
Study characteristics	17	Cite each included study and present its characteristics.	Section 3.1; main study-characteristics table and Supplementary Table S4.
Risk of bias in studies	18	Present assessments of risk of bias for each included study.	Sections 2.7 and 3.7.6; study-level source-grounded assessments in Supplementary Table S5; unclear

Section and Topic	Item #	Checklist item	Location where item is reported
			and not assessable distinguished.
Results of individual studies	19	For all outcomes, present, for each study: (a) summary statistics for each group (where appropriate) and (b) an effect estimate and its precision (e.g. confidence/credible interval), ideally using structured tables or plots.	Sections 3.2–3.6; main results tables and Supplementary Table S4; precision reported where available.
Results of syntheses	20a	For each synthesis, briefly summarise the characteristics and risk of bias among contributing studies.	Sections 3.1–3.7 and 3.7.6; Supplementary Tables S4–S5 (study characteristics and methodological appraisal).
	20b	Present results of all statistical syntheses conducted. If meta-analysis was done, present for each the summary estimate and its precision (e.g. confidence/credible interval) and measures of statistical heterogeneity. If comparing groups, describe the direction of the effect.	Sections 3.2–3.7; descriptive qualitative synthesis and individual study estimates; no meta-analysis.
	20c	Present results of all investigations of possible causes of heterogeneity among study results.	Section 3.7.4; mediator-specific physiological and pre-analytical confounder assessment, Supplementary Table S6.
	20d	Present results of all sensitivity analyses conducted to assess the robustness of the synthesized results.	No formal sensitivity analyses reported.
Reporting biases	21	Present assessments of risk of bias due to missing results (arising from reporting biases) for each synthesis assessed.	No formal synthesis-level missing-results bias assessment; limitations in Section 4.5.
Certainty of evidence	22	Present assessments of certainty (or confidence) in the body of evidence for each outcome assessed.	No formal outcome-level certainty grading reported.
**DISCUSSION**			
Discussion	23a	Provide a general interpretation of the results in the context of other evidence.	Section 4 (Discussion).
	23b	Discuss any limitations of the evidence included in the review.	Section 4.5 (Strengths and Limitations); source-access, assay, matrix and physiological confounding

Section and Topic	Item #	Checklist item	Location where item is reported
			limitations; Supplementary Tables S5–S6.
	23c	Discuss any limitations of the review processes used.	Section 4.5; retrospective OSF registration, incomplete source access and review-process limitations.
	23d	Discuss implications of the results for practice, policy, and future research.	Section 4 (Discussion) and Conclusions; prospective validation and clinical implementation considerations.
**OTHER INFORMATION**			
Registration and protocol	24a	Provide registration information for the review, including register name and registration number, or state that the review was not registered.	Section 2.1: retrospective OSF registration, https://osf.io/2ym5n; registration occurred after review completion.
	24b	Indicate where the review protocol can be accessed, or state that a protocol was not prepared.	Section 2.1: retrospective methodological record at https://osf.io/2ym5n; no publicly deposited prospective protocol established.
	24c	Describe and explain any amendments to information provided at registration or in the protocol.	Section 2.1 and Section 4.5: retrospective registration prevents independent comparison with a prospectively time-stamped protocol; original deviations cannot be verified. Later record amendments, if any, must be disclosed.
Support	25	Describe sources of financial or non-financial support for the review, and the role of the funders or sponsors in the review.	Funding statement; confirm whether funder role is explicitly declared in final manuscript.

Section and Topic	Item #	Checklist item	Location where item is reported
Competing interests	26	Declare any competing interests of review authors.	Conflicts of Interest statement.
Availability of data, code and other materials	27	Report which of the following are publicly available and where they can be found: template data collection forms; data extracted from included studies; data used for all analyses; analytic code; any other materials used in the review.	Data Availability Statement; Supplementary Tables S1–S6. Availability of underlying extraction forms and analytical materials should be stated explicitly.

From: Page MJ, McKenzie JE, Bossuyt PM, Boutron I, Hoffmann TC, Mulrow CD, et al. The PRISMA 2020 statement: an updated guideline for reporting systematic reviews. BMJ 2021;372:n71. doi: 10.1136/bmj.n71 [6]. This work is licensed under CC BY 4.0. To view a copy of this license, visit https://creativecommons.org/licenses/by/4.0/.

Table S2. Complete electronic search strategies and database-specific retrieval results.

Database	Complete search strategy	Date of final search	Restrictions	Records retrieved, n
**PubMed/ MEDLINE**	("Stomach Neoplasms"[Mesh] OR "gastric cancer"[Title/Abstract] OR "gastric cancers"[Title/Abstract] OR "gastric carcinoma"[Title/Abstract] OR "gastric carcinomas"[Title/Abstract] OR "gastric adenocarcinoma"[Title/Abstract] OR "gastric adenocarcinomas"[Title/Abstract] OR "stomach cancer"[Title/Abstract] OR "stomach cancers"[Title/Abstract] OR "stomach carcinoma"[Title/Abstract] OR "stomach carcinomas"[Title/Abstract]) AND ("Neurotransmitter Agents"[Mesh] OR neurotransmitter*[Title/Abstract] OR neuropeptide*[Title/Abstract] OR neuroactive[Title/Abstract] OR serotonin[Title/Abstract] OR "5-hydroxytryptamine"[Title/Abstract] OR dopamine[Title/Abstract] OR norepinephrine[Title/Abstract] OR noradrenaline[Title/Abstract] OR epinephrine[Title/Abstract] OR adrenaline[Title/Abstract] OR metanephrine[Title/Abstract] OR normetanephrine[Title/Abstract] OR "gamma-aminobutyric acid"[Title/Abstract] OR GABA[Title/Abstract] OR galanin[Title/Abstract] OR "substance P"[Title/Abstract] OR tachykinin*[Title/Abstract] OR "neuropeptide Y"[Title/Abstract] OR NPY[Title/Abstract] OR neurotensin[Title/Abstract] OR "vasoactive intestinal peptide"[Title/Abstract] OR VIP[Title/Abstract] OR "pituitary adenylate cyclase-activating polypeptide"[Title/Abstract] OR "PACAP-27"[Title/Abstract] OR "PACAP-38"[Title/Abstract] OR "neuromedin U"[Title/Abstract] OR "cocaine- and amphetamine-regulated transcript"[Title/Abstract] OR "CART peptide"[Title/Abstract] OR somatostatin[Title/Abstract] OR "beta-endorphin"[Title/Abstract] OR endorphin*[Title/Abstract] OR enkephalin*[Title/Abstract] OR "gastrin-	02 September 2026	No publication-date restriction	**1676**

Database	Complete search strategy	Date of final search	Restrictions	Records retrieved, n
	releasing peptide"[Title/Abstract] OR "progastrin-releasing peptide"[Title/Abstract] OR ProGRP[Title/Abstract] OR motilin[Title/Abstract] OR cholecystokinin[Title/Abstract] OR "peptide YY"[Title/Abstract] OR PYY[Title/Abstract] OR obestatin[Title/Abstract] OR "calcitonin gene-related peptide"[Title/Abstract] OR CGRP[Title/Abstract])			
**Scopus**	TITLE-ABS-KEY("gastric cancer" OR "gastric cancers" OR "gastric carcinoma" OR "gastric carcinomas" OR "gastric adenocarcinoma" OR "gastric adenocarcinomas" OR "stomach cancer" OR "stomach cancers" OR "stomach carcinoma" OR "stomach carcinomas") AND TITLE-ABS-KEY(neurotransmitter* OR neuropeptide* OR neuroactive OR serotonin OR "5-hydroxytryptamine" OR dopamine OR norepinephrine OR noradrenaline OR epinephrine OR adrenaline OR metanephrine OR normetanephrine OR "gamma-aminobutyric acid" OR GABA OR galanin OR "substance P" OR tachykinin* OR "neuropeptide Y" OR NPY OR neurotensin OR "vasoactive intestinal peptide" OR VIP OR "pituitary adenylate cyclase-activating polypeptide" OR "PACAP-27" OR "PACAP-38" OR "neuromedin U" OR "cocaine- and amphetamine-regulated transcript" OR "CART peptide" OR somatostatin OR "beta-endorphin" OR endorphin* OR enkephalin* OR "gastrin-releasing peptide" OR "progastrin-releasing peptide" OR ProGRP OR motilin OR cholecystokinin OR "peptide YY" OR PYY OR obestatin OR "calcitonin gene-related peptide" OR CGRP)	02 September 2026	No publication-date restriction	**2455**
**Web of Science Core Collection**	TS=("gastric cancer" OR "gastric cancers" OR "gastric carcinoma" OR "gastric carcinomas" OR "gastric adenocarcinoma" OR "gastric adenocarcinomas" OR "stomach cancer" OR "stomach cancers" OR "stomach carcinoma" OR "stomach carcinomas") AND TS=(neurotransmitter* OR neuropeptide* OR neuroactive OR serotonin OR "5-hydroxytryptamine" OR dopamine OR norepinephrine OR noradrenaline OR epinephrine OR adrenaline OR metanephrine OR normetanephrine OR "gamma-aminobutyric acid" OR GABA OR galanin OR "substance P" OR tachykinin* OR "neuropeptide Y" OR NPY OR neurotensin OR "vasoactive intestinal peptide" OR VIP OR "pituitary adenylate cyclase-activating polypeptide" OR "PACAP-27" OR "PACAP-38" OR "neuromedin U" OR "cocaine- and amphetamine-regulated transcript" OR "CART peptide" OR somatostatin OR "beta-endorphin" OR endorphin* OR enkephalin* OR "gastrin-releasing peptide" OR "progastrin-releasing peptide" OR ProGRP OR motilin OR cholecystokinin OR "peptide YY" OR PYY OR obestatin OR "calcitonin gene-related peptide" OR CGRP)	02 September 2026	No publication-date restriction	**1071**

Database	Complete search strategy	Date of final search	Restrictions	Records retrieved, n
**Total before deduplication**	—	—	—	**5202**

We conducted searches from database inception to 02 September 2026, with no publication-date restriction. We used controlled vocabulary where available and combined it with free-text terms for gastric malignancy and individual neuroactive mediators. No language restriction was incorporated into the electronic search syntax. We also screened the reference lists of eligible articles and relevant reviews, and performed targeted mediator-specific searches when needed to identify potentially missed studies. We did not search Embase because institutional access was unavailable. Abbreviations: CART, cocaine- and amphetamine-regulated transcript; CGRP, calcitonin gene-related peptide; GABA, gamma-aminobutyric acid; GC, gastric cancer; GRP, gastrin-releasing peptide; NPY, neuropeptide Y; PACAP, pituitary adenylate cyclase-activating polypeptide; ProGRP, progastrin-releasing peptide; PYY, peptide YY; TS, Topic; VIP, vasoactive intestinal peptide.

Table S3. Translational evidence mapping of circulating neuroactive mediators in gastric cancer.

Mediator/pathway	Circulating human GC evidence	Clinical direction/ signal	Tumor/ receptor or experimental evidence	Principal receptor/ pathway	Translational interpretation	Major evidence gap
Serotonin (5-HT)	Yes [5,10-13]	Historical whole-blood studies generally reported ↓ 5-HT [10-12]; targeted serum profiling included 5-HT [13], whereas recent plasma metabolomics reported ↑ 5-HT [5]	Strong recent mechanistic evidence links 5-HT signaling to GC biology and ferroptosis resistance [5]	5-HT signaling; TGM2-mediated serotonylation → GPX4 stabilization	Repeatedly investigated across several decades, but findings are strongly dependent on biological matrix and analytical context	Standardized platelet-poor plasma studies; harmonized preanalytics; independent clinical validation
Dopamine	Yes [14]	↓ circulating dopamine in GC versus controls [14]	Altered dopamine-receptor expression reported in GC tumor and peripheral compartments [14]	Dopamine receptors; PKA/ERK-related signaling	Circulating and tumor-associated findings support dysregulation of dopaminergic signaling	Independent circulating replication; prospective clinicopathological and outcome validation
Epinephrine / norepinephrine	No eligible direct circulating parent-catecholamine	—	Adrenergic signaling provides biological plausibility in stress-related and tumor-associated pathways	α-/β-adrenergic receptors	Mechanistically relevant pathway without direct circulating parent-neurotransmitter validation	Standardized direct epinephrine/norepinephrine measurements in GC cohorts

Mediator/pathway	Circulating human GC evidence	Clinical direction/ signal	Tumor/ receptor or experimental evidence	Principal receptor/ pathway	Translational interpretation	Major evidence gap
	measurement identified					
Metanephrine	Yes [15,16]	Higher concentrations associated with more advanced clinicopathological characteristics [15]; exploratory evidence also reported [16]	Reflects catecholamine metabolism and systemic sympathoadrenal activity	Catecholamine metabolic pathway	Candidate systemic marker associated with GC burden rather than direct measurement of epinephrine	Larger cohorts; tumor specificity; multivariable validation
Normetanephrine	Yes [15,16]	Higher concentrations associated with greater tumor burden; adverse survival association reported [15]	Indirect marker of catecholaminergic activity	Catecholamine metabolic pathway	Candidate clinicopathological and prognostic marker	Independent prognostic validation and adjustment for relevant confounders
Galanin (GAL)	Yes, independently replicated [17,18]	↓ in GC versus controls; lower levels associated with greater tumor burden; postoperative increase reported [17]	GAL/GalR signaling has been implicated in tumor-related biology	GalR1–GalR3	Replicated circulating signal with clinicopathological and perioperative associations	Larger prospective cohorts; diagnostic-performance and prognostic validation
Substance P (SP)	Yes [19]	↑ serum SP reported in GC [19]	NK-1R-related experimental evidence supports proliferative, migratory, and invasive effects	SP/NK-1R signaling	Historical circulating observation supported by mechanistic plausibility	Contemporary independent circulating replication using standardized assays
Neuropeptide Y (NPY)	Yes [20]	↓ circulating NPY; lower levels associated with advanced stage, larger tumors and/or weight loss [20]	Y-receptor signaling is relevant to neural, vascular, and metabolic tumor biology	Y-receptor family	Potential marker of cancer burden, although nutritional/metabolic status may substantially influence concentrations	GC-specific replication and adjustment for nutritional status, weight loss, stage, and other confounders
Neurotensin (NT)	Yes [21,22]	Increased circulating NT reported [22]; additional	NT promotes GC invasion and migration	NTSR1 → PKC/ERK/PI3K →	Circulating observations have complementary	Independent circulating replication; standardized

Mediator/pathway	Circulating human GC evidence	Clinical direction/ signal	Tumor/ receptor or experimental evidence	Principal receptor/ pathway	Translational interpretation	Major evidence gap
		peptide-panel evidence demonstrated group-dependent variation [21]	through MMP-9-related signaling [22,23]	MMP-9; EMT-related pathways	mechanistic support from the NT/NTSR1 axis	assays; external diagnostic/prognostic validation
Vasoactive intestinal peptide (VIP)	Yes [19,21]	Measured in GC, but no consistently replicated GC-specific direction across available studies	VIP/VPAC signaling provides biological plausibility in gastrointestinal tumor biology	VPAC1/VPAC2	Clinically heterogeneous circulating evidence despite biological plausibility	Dedicated contemporary GC biomarker studies with standardized sampling
PACAP	No eligible circulating human GC study identified	—	PAC1/VPAC signaling provides mechanistic rationale	PAC1, VPAC1, VPAC2	Mechanistic interest without circulating human GC validation	First targeted circulating GC studies
CGRP	No eligible circulating human GC study identified	—	Neurovascular and neural signaling provides biological rationale	CALCRL/RAMP1 receptor complex	Potential neuroactive pathway without circulating clinical validation	GC-specific tissue-to-circulation translational studies
GABA	No eligible circulating human GC study identified	—	Gastric tumor tissue/cell studies support GABA-related signaling	GABA receptors; MAPK/ERK-related pathways	Mechanistic evidence has not yet translated into a circulating GC biomarker	Targeted circulating human GC studies
Somatostatin	Yes [31]	↑ somatostatin-like immunoreactivity reported in GC [31]	Somatostatin-receptor biology is established in gastrointestinal tissues and tumors	SSTR1–SSTR5	Historical circulating signal with biological plausibility	Replication using contemporary analytical platforms and well-characterized cohorts
β-Endorphin	Yes [19]	↑ serum β-endorphin reported in GC [19]	Opioid signaling provides neuroimmune and tumor-	Opioid receptors	Limited historical circulating evidence requiring cautious interpretation	Contemporary replication controlling for pain, stress, analgesics, and treatment

Mediator/pathway	Circulating human GC evidence	Clinical direction/ signal	Tumor/ receptor or experimental evidence	Principal receptor/ pathway	Translational interpretation	Major evidence gap
			related biological plausibility			
Leucine-enkephalin	Yes [19]	No clear serum difference established [19]	Opioid pathway provides biological context	Opioid receptors	Current evidence does not establish a reproducible circulating GC signal	Contemporary targeted studies with sensitive analytical methods
GRP / ProGRP pathway	Yes for circulating ProGRP [33]	Markedly ↑ ProGRP in GC; associated with disease burden and ↓ after systemic treatment; diagnostic performance reported [33]	GRP/bombesin-family signaling provides mechanistic context	GRPR/bombesin receptor family	Clinically developed single-study signal spanning diagnostic, clinicopathological, and treatment-monitoring domains; ProGRP should not be equated with mature GRP	Independent diagnostic validation; treatment-response replication; prospective outcome studies
Motilin	Yes, independently investigated [19,24,32]	Generally ↑ in GC; postoperative/perioperative changes reported [32]; additional circulating evidence reported in earlier studies [19,24]	Strongly linked to gastrointestinal motility and neuroendocrine physiology	MLNR	Repeated circulating signal, but physiological and surgical determinants complicate tumor-specific interpretation	Standardized fasting studies separating tumor-related effects from gastric motility and postoperative physiology
Cholecystokinin (CCK)	Yes [21,25-27]	↓ CCK in GC versus healthy controls reported in diagnostic studies [21,27]; perioperative CCK responses investigated before and after gastrectomy [25,26]	CCK receptor signaling provides established gastrointestinal biological plausibility	CCK1R/CCK2R	Repeated human investigation supports clinical interest, but diagnostic and perioperative evidence represent different biological contexts; [32] and [33] are overlapping reports rather than	Independent diagnostic validation; standardized fasting and sampling conditions; separation of tumor-related from post-gastrectomy physiological effects

Mediator/pathway	Circulating human GC evidence	Clinical direction/ signal	Tumor/ receptor or experimental evidence	Principal receptor/ pathway	Translational interpretation	Major evidence gap
					independent replication	
Peptide YY (PYY/PYY3-36)	Yes [21,28,29]	Cross-sectional GC-related measurements reported [21]; postoperative PYY3-36 changes demonstrated after gastrectomy [28,29]	Y-receptor-mediated gut–brain signaling provides biological context	Y-receptor family	Evidence currently reflects both cancer-associated measurements and substantial postoperative gastrointestinal adaptation	Dedicated pretreatment GC-control cohorts; standardized longitudinal studies separating tumor effects from surgical physiology
Obestatin	Yes [18,29,30]	↑ in GC versus healthy controls reported [18]; circulating concentrations also change following gastric resection [29,30]	Gut–brain/metabolic signaling provides biological context, although receptor attribution remains incompletely resolved	Neuroendocrine/metabolic signaling; receptor assignment remains uncertain	Evidence now includes both diagnostic case–control and longitudinal perioperative observations, but these contexts should not be interpreted as equivalent	Independent pretreatment replication; mechanistic clarification; standardized longitudinal assessment
Neuromedin U (NMU)	No eligible circulating human GC study identified	—	NMU/NMUR biology provides mechanistic rationale	NMUR1/NMUR2	Mechanistically plausible pathway lacking circulating clinical validation	First circulating human GC studies
CART peptide	No eligible circulating human GC study identified	—	Gastric neuronal/tissue observations provide biological context	Receptor/signaling incompletely defined	Evidence remains predominantly tissue/experimental	Targeted circulating human GC studies

Circulating biomarker evidence refers to eligible human GC studies included in the systematic review. We considered tissue-, receptor-, cellular-, and experimental evidence separately for translational contextualization, and it did not contribute to the clinical study count. Absence of eligible circulating evidence does not imply absence of biological relevance. We did not consider serum, plasma, and whole-blood measurements analytically interchangeable. We did not consider reports from the same or substantially overlapping patient population as independent replications; specifically, we treated references [25] and [26] as overlapping reports. Directional arrows indicate the predominant reported change relative to the relevant comparator and do not imply causality. Symbols: ↑, higher circulating concentration in patients with gastric cancer compared with the relevant control group, or an increase relative to the specified sampling time point; ↓, lower circulating

concentration in patients with gastric cancer compared with the relevant control group, or a decrease relative to the specified sampling time point; →, no statistically significant difference or no consistent directional change. Directional arrows summarize the findings reported by the original studies and do not imply causality. Abbreviations: 5-HT, 5-hydroxytryptamine; CALCRL, calcitonin receptor-like receptor; CART, cocaine- and amphetamine-regulated transcript; CCK, cholecystokinin; CGRP, calcitonin gene-related peptide; EMT, epithelial–mesenchymal transition; GABA, gamma-aminobutyric acid; GAL, galanin; GalR, galanin receptor; GC, gastric cancer; GPX4, glutathione peroxidase 4; GRP, gastrin-releasing peptide; GRPR, gastrin-releasing peptide receptor; MLNR, motilin receptor; MMP-9, matrix metalloproteinase-9; NK-1R, neurokinin-1 receptor; NMU, neuromedin U; NMUR, neuromedin U receptor; NPY, neuropeptide Y; NT, neurotensin; NTSR1, neurotensin receptor 1; PACAP, pituitary adenylate cyclase-activating polypeptide; ProGRP, pro-gastrin-releasing peptide; PYY, peptide YY; RAMP1, receptor activity-modifying protein 1; SP, substance P; SSTR, somatostatin receptor; TGM2, transglutaminase 2; VIP, vasoactive intestinal peptide.

Table S4. Integrated evidence on circulating neuroactive mediators in gastric cancer.

Mediator	Study [Ref.]; GC population	Circulating findings / matrix	Diagnostic evidence	Clinicopathological evidence	Treatment-associated / prognostic evidence
5-HT	Podil'chak et al., 1970 [11]; n NR	Whole blood; Historical altered blood 5-HT; quantitative details not reliably extractable	No validated diagnostic estimate	Not established	Not established
5-HT	Agaev et al., 1973 [12]; n NR	Whole blood; Blood 5-HT investigated in relation to GC and liver function; direction not reliably extractable	No validated diagnostic estimate	Not established	Not established
5-HT	Agaev and Guliev, 1977 [10]; n = 35	Whole blood; ↓ 5-HT in GC; urinary 5-HIAA also assessed	No validated diagnostic estimate	Not established	Not established
5-HT	Choi et al., 2016 [13]; n NR	Serum; Altered tryptophan metabolism; 5-HT quantified in targeted metabolite panel (not isolated diagnostic validation)	No validated diagnostic estimate	Not established	Not established
5-HT	Bai et al., 2026 [5]; n = 41	Plasma; Higher plasma 5-HT; experimental TGM2–GPX4 serotonylation/ferroptosis mechanism	No validated diagnostic estimate	Not established	Not established
DA	Amjadi et al., 2023 [14]; n NR	Plasma; dopamine lower in GC (approximately 1298 vs. 4651 pg/mL); no validated threshold.	No validated diagnostic estimate	Not established	Not established
Metanephrine	Balea et al., 2019 [16];	Serum/plasma; Exploratory metabolite	Not assessed	Exploratory tumor/differentiation assessment in n =	Not established

Mediator	Study [Ref.]; GC population	Circulating findings / matrix	Diagnostic evidence	Clinicopathological evidence	Treatment-associated / prognostic evidence
	n = 4	assessment; no robust comparator		4; no robust subgroup inference.	
Metanephrine	Mehedințeanu et al., 2021 [15]; n = 91	Plasma; Metabolite associated with selected adverse tumor characteristics	Not assessed	T3–4 p = 0.0165; N ≥ 2 p = 0.0473; TNM III–IV p = 0.0148; histological differences nonuniform.	Two-year survival 48.07% (high) vs. 58.33% (low); cutoff 65 pg/mL; HR 1.462; log-rank p = 0.1487 (NS).
Normetanephrine	Balea et al., 2019 [16]; n = 4	Serum/plasma; Exploratory metabolite assessment; no robust comparator	Not assessed	Exploratory tumor/differentiation assessment in n = 4; no robust subgroup inference.	Not established
Normetanephrine	Mehedințeanu et al., 2021 [15]; n = 91	Plasma; Metabolite associated with selected adverse tumor characteristics	Not assessed	Tumor ≥5 cm p = 0.0217; T3–4 p = 0.0177; N ≥ 2 p = 0.0127; TNM III–IV p = 0.0275.	Two-year survival 42.30% (high) vs. 66.67% (low); cutoff 196 pg/mL; HR 2.289; log-rank p = 0.0104; not multivariable-validated.
GAL	Zhang et al., 2019 [17]; n = 34	Plasma; GC 20.73 ± 2.52 vs. controls 36.27 ± 5.94 pg/mL (p < 0.001).	No validated diagnostic estimate	Tumor size r = −0.425; nodes r = −0.309; TNM r = −0.368 (all p < 0.05); invasion depth and differentiation NS.	GAL increased from 20.73 ± 2.52 to 32.74 ± 4.15 pg/mL after resection (p < 0.01); no recurrence/survival validation.
GAL	Uygur et al., 2022 [18]; n = 30	Serum; GC 19.73 ± 5.04 vs. controls 35.59 ± 10.94 pg/mL (p < 0.001).	No validated diagnostic estimate	Not established	Not established
NPY	Li et al., 1998 [20]; GC n = 18; CRC n = 20	Plasma; 125.3 ± 31.5 vs. 145.1 ± 44.1 pg/mL (p < 0.001) in pooled GC/CRC cases versus controls; not GC-only.	No validated diagnostic estimate	GC subgroup: lower with tumor >5 cm (p < 0.05), advanced stage and weight loss >3 kg (p < 0.01); invasion, nodes and grade NS.	Not established
SP / β-endorphin	Xu et al., 1993 [19]; n = 35	Serum; SP and β-endorphin higher in advanced GC (both p < 0.01).	No validated diagnostic estimate	Not established	Not established

Mediator	Study [Ref.]; GC population	Circulating findings / matrix	Diagnostic evidence	Clinicopathological evidence	Treatment-associated / prognostic evidence
Somatostatin	Pawlikowski et al., 1989 [31]; n NR	Peripheral venous blood; ↑ somatostatin-like immunoreactivity in GC	No validated diagnostic estimate	Not established	Not established
Motilin	Feng et al., 1993 [24]; n = 33	Plasma; ↑ plasma motilin in GC	No validated diagnostic estimate	Not established	Not established
Motilin	Zhang et al., 2008; [32]; n = 39	Plasma; ↑ preoperative motilin; ↓ on postoperative day 1 followed by recovery toward preoperative values	No validated diagnostic estimate	Stage II > III ($p < 0.05$), nonmonotonic; not independently replicated.	Motilin decreased on postoperative day 1 ($p < 0.001$), approaching preoperative values by day 7.
Motilin	Xu et al., 1993 [19]; n = 35	Serum; No clear serum difference; gastric juice changes more prominent	No established diagnostic performance	Not established	Not established
CCK	Inoue et al., 1987 [25]; n = 5	Plasma; Meal-stimulated circulating CCK and gallbladder responses evaluated before and after gastrectomy; overlapping with [29]	Not assessed	Not established	CCK/gallbladder responses after gastrectomy; overlapping/complementary reports [28,29], not independent cohorts.
CCK	Huang et al., 1989 [26]*; n = 5	Plasma; Altered CCK release after gastrectomy; overlapping/complementary to Inoue et al. [28]; overlapping with [28]	Not assessed	Not established	CCK/gallbladder responses after gastrectomy; overlapping/complementary reports [28,29], not independent cohorts.
CCK	Akter et al., 2017 [27]; n = 75	Plasma; ↓ circulating CCK in GC; diagnostic discrimination reported	Diagnostic discrimination reported; numerical ROC metrics not verified in source tables.	Not established	Not established
CCK	Zygulska et al., 2019 [21]; n = 20	Fasting plasma; lower CCK in GC ($p = 0.02$); GC n = 20 in mixed GC/CRC study.	No validated diagnostic estimate	Not established	Not established

Mediator	Study [Ref.]; GC population	Circulating findings / matrix	Diagnostic evidence	Clinicopathological evidence	Treatment-associated / prognostic evidence
NT	Akter et al., 2015 [22]; n NR	Plasma; ↑ plasma NT and positive relationship with MMP-9 activity	Diagnostic assessment reported; exact ROC values unverified.	Positive association with MMP-9 activity (biological link); no established TNM association.	Not established
NT	Zygulska et al., 2019 [21]; n = 20	Fasting plasma; NT profiles heterogeneous	No established diagnostic performance	Not established	Not established
VIP / Leu-enkephalin	Xu et al., 1993 [19]; n = 35	Serum; No clear serum difference; gastric juice changes more prominent	No established diagnostic performance	Not established	Not established
VIP / Leu-enkephalin	Zygulska et al., 2019 [21]; n = 20	Fasting plasma; VIP profiles heterogeneous	No established diagnostic performance	Not established	Not established
PYY3-36 / PYY	Kim et al., 2012 [28]; n = 16	Plasma; PYY3-36 ↑ after gastrectomy; postoperative response differed according to vagal preservation	Not assessed	Not established	PYY3-36 increased after surgery; response varied by vagal preservation; no oncological endpoints.
PYY3-36 / PYY	Jeon et al., 2015 [29]; n NR	Circulating blood samples; Perioperative change in PYY3-36	Not assessed	Not established	Short-term PYY3-36 changes after gastric resection.
PYY3-36 / PYY	Zygulska et al., 2019 [21]; n = 20	Fasting plasma; PYY profiles heterogeneous	No established diagnostic performance	Not established	Not established
Obestatin	Kim et al., 2009 [30]; n = 45	Serum; Obestatin changed after gastrectomy, with an early postoperative decrease followed by recovery toward baseline	Not assessed	Not established	Obestatin decreased early after distal gastrectomy, then recovered toward baseline; no oncological endpoints.
Obestatin	Jeon et al., 2015 [29]; n NR	Circulating blood samples; Perioperative change in obestatin	Not assessed	Not established	Short-term obestatin changes after gastric resection.

Mediator	Study [Ref.]; GC population	Circulating findings / matrix	Diagnostic evidence	Clinicopathological evidence	Treatment-associated / prognostic evidence
Obestatin	Uygur et al., 2022 [18]; n = 30	Serum; GC 40.21 ± 5.82 vs. controls 15.15 ± 3.32 ng/mL (p < 0.001).	No validated diagnostic estimate	Not established	Not established
ProGRP	Li et al., 2020 [33]; n = 150	Serum; GC 249.3 ± 28.9; healthy 14.4 ± 7.6; benign gastric disease 20.1 ± 5.9 pg/mL (p < 0.001).	AUC 0.713 (95% CI 0.631–0.795); cutoff 28.3 pg/mL; sensitivity 85.9%, specificity 81.2%. With CEA + CA72-4: 91.2% / 93.4% (Se / Sp); no external validation.	Tumor size, T, N, differentiation and TNM (all p < 0.005); stage III–IV 269.1 ± 30.9 vs. I–II 198.5 ± 23.9 pg/mL; CEA r = 0.680, CA72-4 r = 0.773 (both p < 0.01).	After 3 chemotherapy cycles: reported 249.3 ± 28.9 → 26.1 ± 12.8 pg/mL (p < 0.001); subgroup baseline comparability unconfirmed; no response/survival validation.

Notes. Twenty-four publications represent 23 independent cohorts; Inoue [25] and Huang [26] are overlapping/complementary reports. Study-specific rows may recur when a publication assessed multiple mediators; these do not represent additional publications. GC/CRC pooled NPY case–control data [20] are not GC-specific. Whole-blood, serum, and plasma measurements are not interchangeable. “Not assessed” denotes an outcome not evaluated; “NS” denotes a reported nonsignificant result; absence of a validated diagnostic estimate does not imply a negative diagnostic study. For ProGRP, the reported pre-/post-chemotherapy comparison should not be interpreted as a verified paired baseline for the 108-patient treatment subgroup. ROC estimates for NT and CCK require verification against primary publications. Associations are observational and do not establish independence from confounding. **Abbreviations.** 5-HT, serotonin; AUC, area under the curve; CA72-4, carbohydrate antigen 72-4; CCK, cholecystokinin; CEA, carcinoembryonic antigen; CRC, colorectal cancer; DA, dopamine; GAL, galanin; GC, gastric cancer; HR, hazard ratio; MMP-9, matrix metalloproteinase-9; NPY, neuropeptide Y; NT, neurotensin; PYY, peptide YY; ProGRP, progastrin-releasing peptide; Se, sensitivity; SP, substance P; Sp, specificity; TNM, tumor–node–metastasis; VIP, vasoactive intestinal peptide.

Table S5. Study-level risk-of-bias and methodological quality assessment of publications investigating cir-culating neuroactive mediators in gastric cancer.

Table S5. A. Study-level assessment and source limitations.

Study	Source	Study design / framework	Selection / comparison	Measurement / confounding / timing	Source-grounded assessment and action
Podil'chak et al., 1970 [11]	Bibliographic record only — excluded from included-study RoB denominator	Eligibility cannot be established; appraisal instrument not assignable	NA	NA	Not included in qualitative effect synthesis. Retained here for transparent audit only; no direction-of-effect claim.
Agaev et al., 1973 [12]	Bibliographic record only — excluded from	Eligibility cannot be established;	NA	NA	Not included in qualitative effect synthesis. Retained

	included-study RoB denominator	appraisal instrument not assignable			here for transparent audit only; no direction-of-effect claim.
Agaev and Guliev, 1977 [10]	Abstract	Observational / perioperative; JBI design-specific checklist pending verification	U	Measurement: U; confounding: U; timing: U	Abstract supports blood serotonin assessment in 35 GC cases; methods and adjustment insufficient for a definitive domain rating.
Choi et al., 2016 [13]	Unverified / scan	Observational / perioperative; JBI design-specific checklist pending verification	U	Measurement: U; confounding: U; timing: U	Original clinical report not available in supplied sources; verify actual circulating serotonin result before substantive synthesis.
Bai et al., 2026 [5]	Full text	Observational / perioperative; JBI design-specific checklist pending verification	U	Measurement: U; confounding: U; timing: U	Human plasma measurements are relevant; laboratory and animal findings must not be counted as independent human cohorts.
Amjadi et al., 2023 [14]	Abstract	Observational / perioperative; JBI design-specific checklist pending verification	U	Measurement: U; confounding: U; timing: U	Abstract reports plasma dopamine ELISA; recruitment, assay handling and confounding incompletely documented.
Balea et al., 2019 [16]	Full text (open access; PMC7014983)	Case series; JBI case-series checklist	U	Measurement: U; confounding: U; timing: U	Descriptive four-person case series includes three gastric adenocarcinoma cases and one gastric lymphoma; plasma/serum terminology inconsistent.
Mehedințeanu et al., 2021 [15]	Full text	Observational / perioperative; JBI design-specific	U	Measurement: U; confounding: U; timing: U	Full text supplied; no unverified low-risk judgment

		checklist pending verification			inferred solely from source availability.
Zhang et al., 2019 [17]	Full text	Observational / perioperative; JBI design-specific checklist pending verification	U	Measurement: U; confounding: U; timing: U	Full text supplied; plasma galanin comparison and surgical sampling are assessable, but residual confounding remains a concern.
Uygur et al., 2022 [18]	Full text	Observational / perioperative; JBI design-specific checklist pending verification	U	Measurement: U; confounding: U; timing: U	Full text supplied; evaluate baseline group comparability and analytical controls before assigning low risk.
Li et al., 1998 [20]	Full text	Observational / perioperative; JBI design-specific checklist pending verification	U	Measurement: U; confounding: U; timing: U	Chinese-language original scan supplied; eligibility supported by plasma neuropeptide Y but detailed methods not independently translated.
Xu et al., 1993 [19]	Abstract	Observational / perioperative; JBI design-specific checklist pending verification	U	Measurement: U; confounding: U; timing: U	English abstract supports circulating serum peptide outcomes; gastric juice outcomes must not be conflated with serum.
Pawlikowski et al., 1989 [31]	Abstract	Observational / perioperative; JBI design-specific checklist pending verification	U	Measurement: U; confounding: U; timing: U	Abstract-only source; patient selection, assay methods and confounding not sufficiently reported.
Feng et al., 1993 [24]	Unverified / scan	Observational / perioperative; JBI design-specific checklist pending verification	U	Measurement: U; confounding: U; timing: U	Chinese-language scanned original supplied; plasma motilin reported but detailed methods not independently translated.

Zhang et al., 2008 [32]	Abstract	Observational / perioperative; JBI design-specific checklist pending verification	U	Measurement: U; confounding: U; timing: U	English abstract supports serum gastrin and plasma motilin; pre-/post-operative changes cannot be attributed to cancer alone.
Inoue et al., 1987 [25]	Full text	Observational / perioperative; JBI design-specific checklist pending verification	U	Measurement: U; confounding: U; timing: U	Full text supplied; physiological response after surgery is not diagnostic-test evidence.
Huang et al., 1989 [26] *	Abstract	Observational / perioperative; JBI design-specific checklist pending verification	U	Measurement: U; confounding: U; timing: U	Abstract supports CCK release/gallbladder physiology; overlap with Inoue 1987 is unconfirmed, so independent-cohort count remains provisional.
Akter et al., 2017 [27]	Abstract	Diagnostic accuracy; QUADAS-2	H: known-case/control sampling	Measurement: U; confounding: U; timing: U	Known GC cases versus healthy controls support high selection bias for diagnostic performance; cut-off pre-specification and independent validation not verifiable.
Zygulska et al., 2019 [21]	Full text	Observational / perioperative; JBI design-specific checklist pending verification	U	Measurement: U; confounding: U; timing: U	Full text supplied; small GC subgroup and multiple comparisons restrict precision.
Akter et al., 2015 [22]	PubMed abstract (PMID 25724188)	Observational / perioperative; JBI design-specific checklist pending verification	U	Measurement: U; confounding: U; timing: U	Abstract supports human plasma neurotensin; in vitro MMP-9 findings are not independent clinical biomarker evidence.

Kim et al., 2012 [28]	Full text	Observational / perioperative; JBI design-specific checklist pending verification	U	Measurement: U; confounding: U; timing: U	Full text supplied; surgical procedure and time since surgery are potential confounders.
Jeon et al., 2015 [29]	Abstract	Observational / perioperative; JBI design-specific checklist pending verification	U	Measurement: U; confounding: U; timing: U	Abstract-only postoperative study; short-term timing and clinical covariates inadequately described.
Kim et al., 2009 [30]	Full text	Observational / perioperative; JBI design-specific checklist pending verification	U	Measurement: U; confounding: U; timing: U	Full text supplied; postsurgical obestatin/ghrelin ratio is not evidence of standalone GC diagnostic performance.
Li et al., 2020 [33]	Full text	Diagnostic accuracy; QUADAS-2	H: known-case/control sampling	Measurement: U; confounding: U; timing: U	Full text supplied; case–control recruitment raises patient-selection/applicability concerns for diagnostic accuracy; threshold validation requires separate review.

Legend: H = high risk of bias supported by available information; U = unclear because available reporting is insufficient; NA = not assessable from bibliographic record. The source-access category is not itself a bias judgment. Diagnostic accuracy: QUADAS-2. Other designs: JBI design-specific appraisal, with only source-supported judgments shown. No claim of complete JBI signaling-question assessment is made.

Table S5. B. Diagnostic accuracy assessment (QUADAS-2).

Study	Patient selection	Index test	Reference standard	Flow / timing	Applicability / justification
Akter et al., 2017 [27]	H (case–control sampling)	U	U	U	U — 75 GC cases versus 36 healthy controls; reported sensitivity and specificity; cutoff prespecification, blinding, and validation cannot be verified.
Li et al., 2020 [33]	H (case–control sampling)	U	U	U	U — 150 GC, 50 healthy and 66

				benign gastric disease controls; diagnostic accuracy requires independent verification of thresholds, reference standard, and flow.

Eligibility, interpretation and reporting restrictions: Podil'chak (1970) [11] and Agaev (1973) [12]: title-only historical records. Eligibility and outcomes are unverified; these records are not counted among eligible studies or independent cohorts. Balea (2019) [16] and Akter (2015) [22] remain eligible for descriptive human circulating-mediator evidence. Balea has only three gastric adenocarcinoma cases among four patients; Akter includes both human circulating neurotensin and separate cell-line experiments. Feng (1993) [24] and Li (1998) [20] are Chinese-language scans. They provide stronger source access than abstract-only records, but a verified translation of methods is needed before issuing affirmative low-risk judgments. Inoue (1987) [25] and Huang (1989) [26]: potential cohort overlap cannot be resolved from accessible information. Do not claim an exact independent-cohort total without source verification. No numerical aggregate quality score or overall percentage of low-risk studies is calculated, because heterogeneous designs and inaccessible reporting would make such a figure misleading.

Table S6. Mediator-Specific Physiological and Pre-analytical Confounders, Reporting Completeness, and Implications for Clinical Interpretation in Gastric Cancer. Mediator-Specific Physiological and Pre-analytical Confounders: Source-Confirmed Reporting and Clinical Interpretation. Evidence base: 22 publication records from Supplementary Tables S4–S5 with potentially assessable circulating-mediator evidence. The two bibliographic-only historical records (Podil'chak et al., 1970[11]; Agaev et al., 1973 [12]) are excluded. This table documents confirmed reporting in accessible source material; it does not establish absence of control when information could not be verified.

Table S6. A. Publication-level evidence extraction.

Publication	Mediator(s)	Fasting / meal status	Specimen processing / storage	Surgical or sampling context
**Agaev & Guliev 1977 [10]**	5-HT	NRV	NRV	NRV
**Choi 2016 [13]**	5-HT	NRV	NRV	NRV
**Bai 2026 [5]**	5-HT	NRV	two-stage plasma centrifugation; storage −80 °C	NRV
**Amjadi 2023 [14]**	DA	NRV	NRV	NRV
**Balea 2019 [16]**	Metanephrines	NRV	NRV	NRV
**Mehedințeanu 2021 [15]**	Metanephrines	fasted; alcohol/caffeine avoided 24 h	prechilled EDTA tubes; cooling	gastrectomy; interfering medication discussed
**Zhang 2019 [17]**	GAL	fasting pre/post blood	prechilled EDTA/aprotinin; centrifugation within 30 min	pre/postoperative samples
**Uygur 2022 [18]**	GAL / obestatin	NRV	NRV	NRV
**Li 1998 [20]**	NPY	NRV	NRV	NRV

**Xu 1993 [19]**	SP / beta-endorphin / motilin / VIP / Leu-enkephalin	fasting blood	NRV	NRV
**Pawlikowski 1989 [31]**	Somatostatin	NRV	NRV	NRV
**Feng 1993 [24]**	Motilin	NRV	NRV	NRV
**Zhang 2008 [32]**	Motilin	NRV	NRV	preoperative; postoperative days 1, 7
**Inoue 1987 [25]**	CCK	14 h fast; standardized test meal	NRV	before and 1 month after gastrectomy
**Huang 1989 [26]**	CCK	NRV	NRV	after gastrectomy
**Akter 2017 [27]**	CCK	NRV	NRV	NRV
**Zygulska 2019 [21]**	CCK / NT / VIP / PYY	fasting plasma	EDTA/aprotinin; storage temperatures reported	NRV
**Akter 2015 [22]**	NT	NRV	NRV	NRV
**Kim 2012 [28]**	PYY	fasted state	NRV	subtotal gastrectomy; vagal preservation
**Jeon 2015 [29]**	PYY / obestatin	NRV	NRV	preoperative; 1 h and postoperative days 1, 3, 7
**Kim 2009 [30]**	Obestatin	NRV	NRV	after distal gastrectomy
**Li 2020 [33]**	ProGRP	NRV	centrifugation 2000 × g for 10 min; −80 °C storage	NRV

NRV = not reliably verified from the accessible source, not proof that the variable was not reported or controlled in the full study. Descriptions are condensed; refer to original methods for implementation details.

Table S6. B. Mediator-specific minimum confirmed reporting frequencies.

Mediator	Publication N	Fasting / meal documented	Handling / storage documented	Surgery / sampling context documented
**5-HT**	3	≥0/3	≥1/3	≥0/3
**DA**	1	≥0/1	≥0/1	≥0/1
**Metanephrine / normetanephrine**	2	≥1/2	≥1/2	≥1/2
**GAL**	2	≥1/2	≥1/2	≥1/2
**NPY**	1	≥0/1	≥0/1	≥0/1
**SP / β-endorphin**	1	≥1/1	≥0/1	≥0/1
**Somatostatin**	1	≥0/1	≥0/1	≥0/1

**Motilin**	3	≥1/3	≥0/3	≥1/3
**CCK**	4	≥2/4	≥1/4	≥2/4
**NT**	2	≥1/2	≥1/2	≥0/2
**VIP / Leu-enkephalin**	2	≥2/2	≥1/2	≥0/2
**PYY**	3	≥2/3	≥1/3	≥2/3
**Obestatin**	3	≥0/3	≥0/3	≥2/3
**ProGRP**	1	≥0/1	≥1/1	≥0/1

Interpretation: each ≥x/N is a minimum confirmed count among N publication records for that mediator, not a verified complete reporting prevalence. Counts are not independent-cohort frequencies. The surgical-context column denotes explicit description of surgery or perioperative/clinical timing; it does not establish control of surgery as a confounder. Fasting/meal and handling fields likewise document description, not protocol adequacy.

Table S6. C. Mediator-specific confounding and clinical implications.

Mediator/class	Priority physiological and pre-analytical variables	Implication for clinical robustness
**Serotonin (5-HT)**	Platelet count and activation, clotting, matrix (whole blood/serum/plasma), centrifugation and residual platelets	Matrix-dependent 5-HT concentrations cannot be pooled as equivalent circulating exposure; plasma centrifugation alone does not verify platelet depletion.
**Catecholaminergic mediators**	Posture, stress, caffeine/alcohol, medications, sampling time and matrix	Uncontrolled physiological conditions can alter circulating metabolite levels independently of tumor status.
**Motilin and CCK**	Fasting/feeding, meal stimulation, migrating motor complex, gallbladder and gastric physiology, gastrectomy	Postoperative trajectories may reflect altered digestive physiology rather than tumor response.
**PYY and obestatin**	Fasting, nutritional status, gastric reconstruction, vagal preservation and postoperative interval	Perioperative peptide shifts are not validated cancer-specific response markers.
**Other neuropeptides (GAL, NPY, NT, VIP, SP, SOM)**	Proteolysis, anticoagulant and protease inhibitors, processing delay, storage and freeze–thaw	Differences in peptide recovery and assay performance restrict cross-study comparisons.
**ProGRP**	Renal function, sample handling, assay platform and diagnostic case–control selection	Reported ROC thresholds require independent validation; potential nonmalignant influences require evaluation.

Source and denominator qualifications: S4 and S5 do not provide exhaustive primary-methods data for every study; full-text verification is incomplete for some abstract-only or untranslated sources. NRV must not be recoded as "not reported" without inspecting the complete original report. Inoue et al. (1987) [25] and Huang et al. (1989) [26] may describe overlapping/complementary CCK cohorts; the 4-publication denominator is not a 4-independent-cohort denominator. A single study can contribute to several mediator categories. The 22-publication base is not the sum of mediator denominators. The counts above reflect the source-confirmed register used in this revision and are suitable for cautious descriptive reporting only. They must not be presented as definitive percentages of pre-analytical compliance.
